# Autoantibody and T cell responses to oxidative post-translationally modified insulin neoantigenic peptides in type 1 diabetes

**DOI:** 10.1007/s00125-022-05812-4

**Published:** 2022-10-07

**Authors:** Rocky Strollo, Chiara Vinci, Y. K. Stella Man, Sara Bruzzaniti, Erica Piemonte, Ghadeer Alhamar, Silvia Irina Briganti, Ilaria Malandrucco, Flavia Tramontana, Chiara Fanali, James Garnett, Roberto Buccafusca, Perrin Guyer, Mark Mamula, Eddie A. James, Paolo Pozzilli, Johnny Ludvigsson, Paul G. Winyard, Mario Galgani, Ahuva Nissim

**Affiliations:** 1 Department of Science and Technology for Humans and the Environment, Università Campus Bio-Medico di Roma, Rome, Italy; 2 Biochemical Pharmacology, William Harvey Research Institute, Queen Mary University of London, London, UK; 3 Institute for Experimental Endocrinology and Oncology ‘G. Salvatore’, Consiglio Nazionale delle Ricerche, Naples, Italy; 4 Department of Biology, Università degli Studi di Napoli ‘Federico II’, Naples, Italy; 5 Department of Molecular Medicine and Medical Biotechnology, Università degli Studi di Napoli ‘Federico II’, Naples, Italy; 6 Department of Medicine, Unit of Endocrinology & Diabetes, Università Campus Bio-Medico di Roma, Rome, Italy; 7 The UOSD of Endocrinology and Metabolic Diseases, Azienda Sanitaria Locale (ASL) Frosinone, Frosinone, Italy; 8 Centre for Host-Microbiome Interactions, Dental Institute, King’s College London, London, UK; 9 School of Biological and Chemical Sciences, Queen Mary University of London, London, UK; 10 Program for Translational Immunology, Benaroya Research Institute, Seattle, WA USA; 11 Department of Medicine, Yale University School of Medicine, New Haven, CT USA; 12 Division of Pediatrics, Department of Biomedical and Clinical Sciences, Crown Princess Victoria Children’s Hospital, Linköping University, Linköping, Sweden; 13 Institute of Biomedical and Clinical Science, University of Exeter Medical School, St Luke’s Campus, Exeter, UK

**Keywords:** Autoimmunity, Immune response, Insulin, Insulin autoantibodies, Insulin neoepitope peptide, Neoantigen, Neoepitope, Oxidative post-translational modifications, Post-translational modifications

## Abstract

**Aims/hypothesis:**

Antibodies specific to oxidative post-translational modifications (oxPTM) of insulin (oxPTM-INS) are present in most individuals with type 1 diabetes, even before the clinical onset. However, the antigenic determinants of such response are still unknown. In this study, we investigated the antibody response to oxPTM-INS neoepitope peptides (oxPTM-INSPs) and evaluated their ability to stimulate humoral and T cell responses in type 1 diabetes. We also assessed the concordance between antibody and T cell responses to the oxPTM-INS neoantigenic peptides.

**Methods:**

oxPTM-INS was generated by exposing insulin to various reactive oxidants. The insulin fragments resulting from oxPTM were fractionated by size-exclusion chromatography further to ELISA and LC-MS/MS analysis to identify the oxidised peptide neoepitopes. Immunogenic peptide candidates were produced and then modified in house or designed to incorporate in silico-oxidised amino acids during synthesis. Autoantibodies to the oxPTM-INSPs were tested by ELISA using sera from 63 participants with new-onset type 1 diabetes and 30 control participants. An additional 18 fresh blood samples from participants with recently diagnosed type 1 diabetes, five with established disease, and from 11 control participants were used to evaluate, in parallel, CD4^+^ and CD8^+^ T cell activation by oxPTM-INSPs.

**Results:**

We observed antibody and T cell responses to three out of six LC-MS/MS-identified insulin peptide candidates: A:12–21 (SLYQLENYCN, native insulin peptide 3 [Nt-INSP-3]), B:11–30 (LVEALYLVCGERGFFYTPKT, Nt-INSP-4) and B:21–30 (ERGFFYTPKT, Nt-INSP-6). For Nt-INSP-4 and Nt-INSP-6, serum antibody binding was stronger in type 1 diabetes compared with healthy control participants (*p*≤0.02), with oxidised forms of ERGFFYTPKT, oxPTM-INSP-6 conferring the highest antibody binding (83% binders to peptide modified in house by hydroxyl radical [^●^OH] and >88% to in silico-oxidised peptide; *p*≤0.001 vs control participants). Nt-INSP-4 induced the strongest T cell stimulation in type 1 diabetes compared with control participants for both CD4^+^ (*p*<0.001) and CD8^+^ (*p*=0.049). CD4^+^ response to oxPTM-INSP-6 was also commoner in type 1 diabetes than in control participants (66.7% vs 27.3%; *p*=0.039). Among individuals with type 1 diabetes, the CD4^+^ response to oxPTM-INSP-6 was more frequent than to Nt-INSP-6 (66.7% vs 27.8%; *p*=0.045). Overall, 44.4% of patients showed a concordant autoimmune response to oxPTM-INSP involving simultaneously CD4^+^ and CD8^+^ T cells and autoantibodies.

**Conclusions/interpretation:**

Our findings support the concept that oxidative stress, and neoantigenic epitopes of insulin, may be involved in the immunopathogenesis of type 1 diabetes.

**Graphical abstract:**

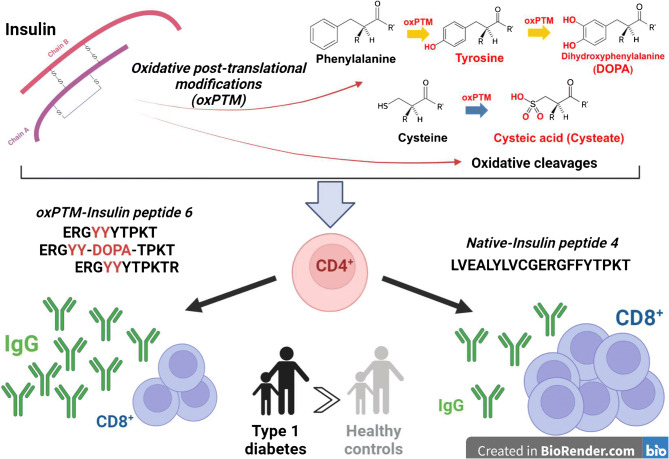

**Supplementary Information:**

The online version contains peer-reviewed but unedited supplementary material available at 10.1007/s00125-022-05812-4.

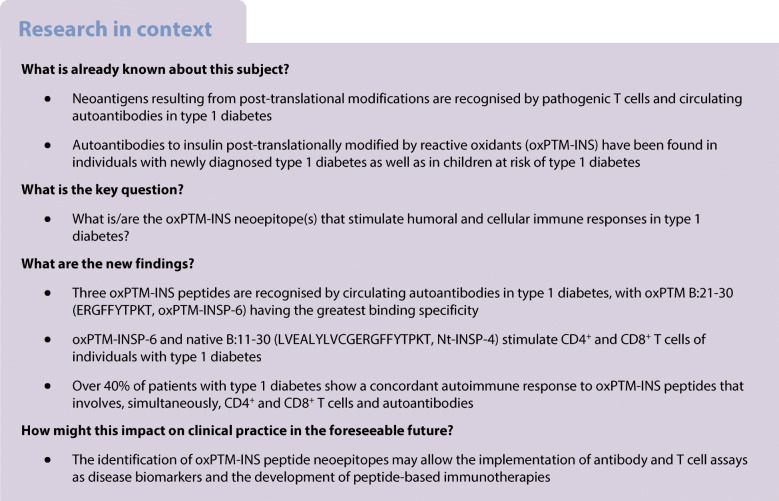



## Introduction

Type 1 diabetes is an organ-specific autoimmune disease resulting from the chronic autoimmune-mediated destruction of insulin-producing pancreatic beta cells [1, 2]. The presence of autoantibodies specific for two or more islet antigens is a reliable predictor of disease progression. Autoantibodies to insulin (IAA), GAD (GADA), tyrosine phosphatase-like molecule IA-2 (IA-2A) and zinc transporter 8 protein (ZnT8A) are early indicators of the loss of tolerance. The greater the number of different islet autoantibodies, the greater an individual’s risk of developing type 1 diabetes [3, 4]. Nevertheless, the mechanism that induces the autoimmune response is not yet fully understood.

Alongside the humoral autoimmune response, CD4^+^ and CD8^+^ T cells are thought to be primarily responsible for beta cell destruction. T cells have been shown to infiltrate pancreatic islets [5, 6] and recognise multiple beta cell antigens [7] such as proinsulin peptides [8]. Neoepitopes recognised by pathogenic T cells are increasingly appreciated but incompletely defined [9]. Neoepitopes are formed when self-proteins undergo post-translational modifications (PTM) to create a new epitope that is selectively recognised by T or B cells as non-self or is more effectively processed, presented and recognised in its modified form.

Beta cell-specific T cell neoepitopes include one, or a combination, of the following: (1) modifications arising during antigen processing and presentation [10]; (2) conversion of glutamine to glutamate, or arginine to citrulline of GAD65 peptides and 78-kDa glucose-regulated protein, GRP78 [11, 12]; (3) peptide fusion [13, 14]; (4) aberrant mRNA translation [15]; (5) a vicinal disulphide bond region within the oxidised insulin alpha-chain [16]; and (6) deamidated tyrosine phosphatase-related IA-2 recognised by T cells in the context of *HLA-DQ8* [17].

Oxidative PTM (oxPTM) by reactive oxidants represents another mechanism for the generation of neoantigenic epitopes. Our previous work demonstrated that most individuals with type 1 diabetes [18] or children at risk of diabetes [19] have autoantibodies to oxidative post-translationally modified insulin (oxPTM-INS). Hence, antibodies to oxPTM-INS can identify those who are negative to the standard islet autoantibodies [18, 20].

Here, we performed a full characterisation of oxPTM-INS and evaluated antibody and T cell responses towards insulin peptides (INSPs) generated by oxPTM in individuals with type 1 diabetes.

## Methods

### Study design

**Fig. 1:**
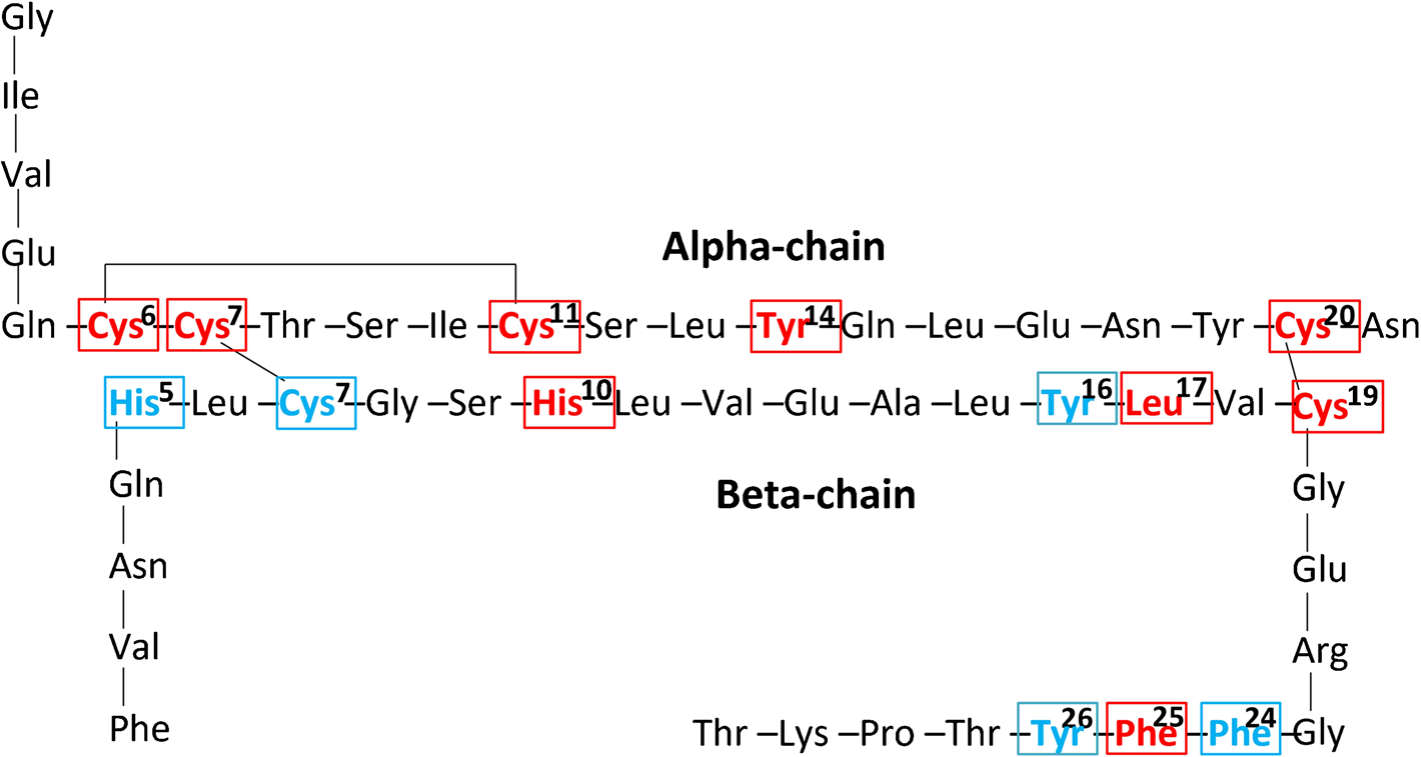
Mapping the oxidised amino acid hotspots in oxPTM-INS. The analyses showed that the main hotspots in oxPTM-INS involved His^5^, Cys^7^, His^10^, Tyr^16^, Leu^17^, Cys^19^, Phe^24^, Phe^25^ and Tyr^26^ in the insulin beta-chain. Additional hotspots were identified in the alpha-chain: Cys^6^, Cys^7^, Cys^11^, Tyr^14^ and Cys^20^. Red boxes indicate the newly discovered oxidation hotspots and blue boxes indicate previously described amino acid hotspots

oxPTM-INS was generated in vitro by exposing human recombinant insulin to reactive oxidants. Size-exclusion chromatography (ÄKTA, UK) in combination with ELISA was employed to analyse the oxPTM-INS profile and to identify immunogenic fractions resulting from the oxPTM further to LC-MS/MS. Peptides discovered by ÄKTA/ELISA/LC-MS/MS were made and exposed to reactive oxidants, to generate oxPTM-INS peptides (oxPTM-INSPs). We also synthesised oxPTM-INSP derivatives designed in silico with oxidised amino acids such as dihydroxyphenylalanine (DOPA) instead of tyrosine, cysteate instead of cysteine or tyrosine instead of phenylalanine. Autoantibodies to oxPTM-INSPs (in house modified and in silico derivatives) were tested using sera from our biobanks of new-onset type 1 diabetes (Study cohort 1). For T cell stimulation we collected fresh blood samples from type 1 diabetes patients (Study cohort 2) to evaluate, in parallel, CD4^+^ and CD8^+^ T cell and autoantibody responses to the oxPTM-INSPs (electronic supplementary material [ESM] Fig. 1).

### Patient cohorts

Type 1 diabetes was diagnosed according to ADA criteria, and in most cases diagnosis was confirmed by islet autoantibodies.

#### Study cohort 1

Serum samples were obtained from the following biobanks: (1) Linköping University (*n*=50), including sera from young patients at 10 days after type 1 diabetes diagnosis, under insulin therapy for 10 days; (2) the Immunotherapy of Diabetes (IMDIAB) cohort (*n*=13), including sera from young individuals with newly diagnosed type 1 diabetes collected before insulin therapy. Thirty age- and sex-comparable non-diabetic participants were used as control participants (Table 1).

**Table 1:** Clinical and biochemical features of the study populations

Variable	Type 1 diabetes	Healthy control participants
Study cohort 1		
*N*	63	30
Age, years	11±4.9	13.8±0.69
Male, *n* (%)	33 (52.4)	10 (33.3)
C-peptide, nmol/l	0.132±0.129	NA
GADA^+^, *n*/*n* (%)	33/42 (78.6)	NA
IA-2A^+^, *n*/*n* (%)	35/43 (81.4)	NA
Study cohort 2		
*N*	18	11
Age, years*	18.75±12.48	29.7±8.59
Male, *n* (%)	8 (44.4)	3 (27.3)
BMI, kg/m^2^	20.00±4.29	22.00±5.31
Children, *n* (%)	5 (27.8)	2 (18.2)
Adults, *n* (%)	14 (77.8)	9 (81.8)
Insulin-naive, *n* (%)	5 (27.8)	NA
Disease duration, years	1.07±3.08	NA
New-onset, <1 month, *n* (%)	5 (27.8)	NA
Duration >1 month and <2 years, *n* (%)	13 (72.2)	NA
HbA_1c_, mmol/mol	74.2±0.87	NA
HbA_1c_, %	8.94±2.23	NA
C-peptide (nmol/l)	0.21±0.13	NA

Data are mean ± SD or *n* (%)

The table shows features related to serum samples of: (1) Study cohort 1 used for the neoantigenic peptide discovery experiments, collected at the Linköping and IMDIAB-Rome biobanks; and (2) Study cohort 2 tested in the T cell stimulation experiments, newly recruited in Rome, Università Campus Bio-Medico, and Naples, Università Federico II

Categorical analyses were performed by Χ^2^ test

*p=0.0498

NA, not applicable

#### Study cohort 2

Fresh blood samples were collected from 18 individuals with type 1 diabetes: 13 adults with disease duration ≤2 years, and five newly diagnosed children naive to insulin treatment. Eleven non-diabetic participants were used as control participants. Blood samples were collected at Università Campus Bio-Medico (Rome, Italy) and Università Federico II (Naples, Italy) (Table 1).

#### Study cohort 3

Fresh blood samples were from five adult participants with type 1 diabetes with disease duration between 2 and 10 years (ESM Table 1).

#### Ethic and consent

The ethical committees at Università Campus Bio-Medico, Rome, Italy; Università Federico II, Naples, Italy; Linköping University, Linköping, Sweden; and Benaroya Research Institute, Seattle, WA, USA have approved the use of blood samples for research, with informed consent signed by the participants or their parents/caregivers.

### Insulin modifications

The insulin samples used for epitope mapping were from two different sources: (1) human recombinant insulin from Sigma (Haverhill, UK; product code no. I2643); and (2) human recombinant insulin Humulin R (Eli Lilly, Italy). Sigma insulin was dissolved in PBS (1 mg/ml) while Humulin R was formulated by the manufacturer at a concentration of 3.47 mg/ml. Insulin was chemically modified as previously described [18] while testing a range of oxidation conditions with NaOCl (hypochlorous acid [HOCl] modification; BDH, Oxford, UK) and/or with CuCl_2_ (Sigma) plus hydrogen peroxide (hydroxyl radical [^●^OH] modification; Sigma) to further optimise modifications.

### ÄKTA pure protein purification

Size-exclusion chromatography (ÄKTA purifier system) was used to fractionate the various insulin fragments obtained from oxPTM. Superdex 30 increase column (GE Healthcare, UK) was suitable for the detection of low molecular weights (100–7000 kDa). Chromatographic profiles at absorbance wavelength 280 nm were recorded for both native insulin (Nt-INS) and oxPTM-INS.

### ELISA for autoantibody detection

The ELISA for Nt-INS and oxPTM-INS autoantibodies was performed as previously described [18] (ESM Methods, ELISA assay for antibody detection).

### Mass spectrometry

Fractions that showed reactivity in ELISA were dried and resuspended in 30 μl of 0.1% formic acid. Fractions were analysed by LC-MS/MS (Orbitrap Velos, Thermo Scientific, UK) at the Cambridge University UK core facility. The analysis was based on the cleaved peptides following oxidation producing singly charged ions, which are not ordinarily selected for tandem MS in a typical proteomic experiment usually digested with specific enzymes and resulting in well-defined peptide cleavage. The insulin breakdown was dependent on oxidation, whereby cleavage sites were less well defined and more as a result of random events.

Analysis of insulin synthetic peptides (produced by Peptide Protein Research, UK; ESM Methods, Peptides synthesis modification assessment) was done by ultra-performance LC coupled with an electrospray ionisation quadrupole time-of-flight mass spectrometer operating in MS exponential (MS^e^) mode (UPLC-qTof/MS^e^), which was used to identify all peptides, and to generate fragment ions upon collision-induced dissociation (CID) to positively confirm their sequences (ESM Methods, Peptide fractionation and mass spectrometry). Structural changes induced by oxPTM were studied by circular dichroism data analysis using Beta Structure Selection (BeStSel server, https://bestsel.elte.hu/) [21] (ESM Methods, Structural changes analysis by circular dichroism).

### In vitro peptide stimulation and T cell proliferation assay

Peripheral blood mononuclear cells (PBMCs) were freshly isolated from type 1 diabetic and healthy individuals using Ficoll-Hypaque density gradient centrifugation. PBMCs were labelled with the fluorescent dye CellTrace Violet (Invitrogen, UK; Thermo Fisher Scientific, UK) and cultured (2×10^5^ cells/well) in round-bottom 96-well plates (Falcon, Becton Dickinson) with RPMI-1640 medium (Gibco, UK; Thermo Fisher Scientific) supplemented with 5% autologous plasma in the presence or not of INSPs (20 ug/ml); purified protein derivative (PPD, 10 ug/ml) and anti-CD3 (0.1 ug/ml; clone OKT3, BD Pharmingen, USA) were used as positive controls. Two scrambled peptides, DNRDGNVYYF and GRKAETELLVYPTCVYLFFG, and the Exendin 9-39 fragment were used as negative controls. After 7 days, PBMCs were stained with PE-Cyanine7 (PE-Cy7) anti-CD8 (clone RPA-T8, BD Pharmingen) and FITC anti-CD3 (clone UCHT1, BD Pharmingen). Samples were analysed using a FACSCanto II (BD Biosciences, UK) to evaluate T cell proliferation measured as CellTrace Violet dilution. Cytofluorimetric analyses were performed using FlowJo software (FlowJo). The results were given as stimulation index (SI), calculated as percentage of stimulated T cell subset proliferation/percentage of unstimulated T cell subset proliferation. The assay for detection of peptide-specific T cell subsets was done as previously described [22] (ESM Methods, Assay for detection of peptide-specific T cells).

### Statistical analyses

Statistical analyses were performed using Prism 9.0 software (GraphPad, San Diego, CA, USA). Cut-off points of positivity (binders) in the antibody ELISA for each peptide were defined by the mean absorbance of healthy control samples to the corresponding native insulin peptide (Nt-INSP) plus 3×SEM. Specificity and sensitivity were evaluated by receiver operating characteristic (ROC) curve analysis. AUC is reported as absolute value and was tested for equality according to DeLong et al [23]. Differences in antibody levels and T cell SIs between groups were tested by one-way ANOVA or Student’s *t* tests, as appropriate. Correlation analyses were tested by Pearson or Spearman’s test, as appropriate. Categorical analyses were performed by χ^2^ or McNemar’s tests, as appropriate. For each set of experiments, *p* values were adjusted for multiple comparisons using the Holm–Sidak test. Hierarchical cluster analysis and principal component analysis (PCA) were done using Clustvis software (https://biit.cs.ut.ee/clustvis/) and Prism 9.0 software, respectively.

## Results

### Mapping of the oxidised amino acid hotspots in the oxPTM-INS

For the epitope mapping, we used multiple size-exclusion chromatography (ÄKTA), ELISA and LC-MS/MS experiments for Sigma insulin and Humulin R insulin. We first confirmed that the reactivity pattern of type 1 diabetes samples to Humulin R oxPTM-INS was similar to Sigma oxPTM-INS (ESM Fig. 2). Size-exclusion chromatography fractions of oxPTM-INS corresponding to small insulin fragments resulting from oxPTM were collected and analysed by ELISA. Fractions that showed reactivity by ELISA were dried and analysed by LC-MS/MS (ESM Fig. 3, ESM Table 2). We have previously reported that amino acids His^5^, Cys^7^, Tyr^16^, Phe^24^ and Tyr^26^ in the beta-chain are oxidised hotspots [18]. In the current study, additional new oxidised amino acid modification hotspots were discovered: His^10^, Leu^17^, Cys^19^ and Phe^25^ of the beta-chain and Cys^6^, Cys^7^, Cys^11^, Tyr^14^ and Cys^20^ of the alpha-chain. Oxidation of Cys^6^ in the alpha-chain was also seen in the Nt-INS (Fig. 1).

LC-MS/MS experimental data mapped neoepitopes to six potential oxPTM-INSPs that span both insulin alpha- and beta-chains. Candidate INSPs included SLYQLENYCN (A:12–21, INSP-3) from the alpha-chain and an additional five peptides from the beta-chain: YLVCGERGFF (B:16–25, INSP-1), LVEALYLVCGER (B:11–22, INSP-2), LVEALYLVCGERGFFYTPKT (B:11–30, INSP-4), FVNQHLC (B:1–7, INSP-5), ERGFFYTPKT (B:21–30, INSP-6). We also included another version of INSP-6 with the addition of a C-terminal arginine (R), as this sequence was seen in several MS profiles and R is the amino acid in the junction with the proinsulin C-peptide (Table 2).

**Table 2:** Six oxidative insulin neoantigenic peptides

INSP	Sequence		Oxidised amino acid hotspots
INSP-1	B:16–25	YLVCGERGFF	Y^16^; L^17^; C^19^; F^24^; F^25^
INSP-2	B:11–22	LVEALYLVCGER	Y^16^; L^17^; C^19^
INSP-3	A:12–21	SLYQLENYCN	Y^14^; C^20^
INSP-4	B:11–30	LVEALYLVCGERGFFYTPKT	Y^16^; L^17^; C^19^; F^24^; F^25^; Y^26^
INSP-5	B:1–7	FVNQHLC	H^5^; C^7^
INSP-6	B:21–30	ERGFFYTPKT	F^24^; F^25^; Y^26^
INSP-6+ R	B:21–31	ERGFFYTPKTR	F^24^; F^25^; Y^26^

List of INSP candidates that were detected by LC-MS/MS following insulin oxPTM. The table highlights the aminoacidic modifications detected by LC-MS/MS that are graphically represented in Fig. 1

INSP-6+ R, INSP-6 plus R at the C-terminal end

### Antibody reactivity of type 1 diabetes serum against the candidate oxPTM-INSPs

**Fig. 2:**
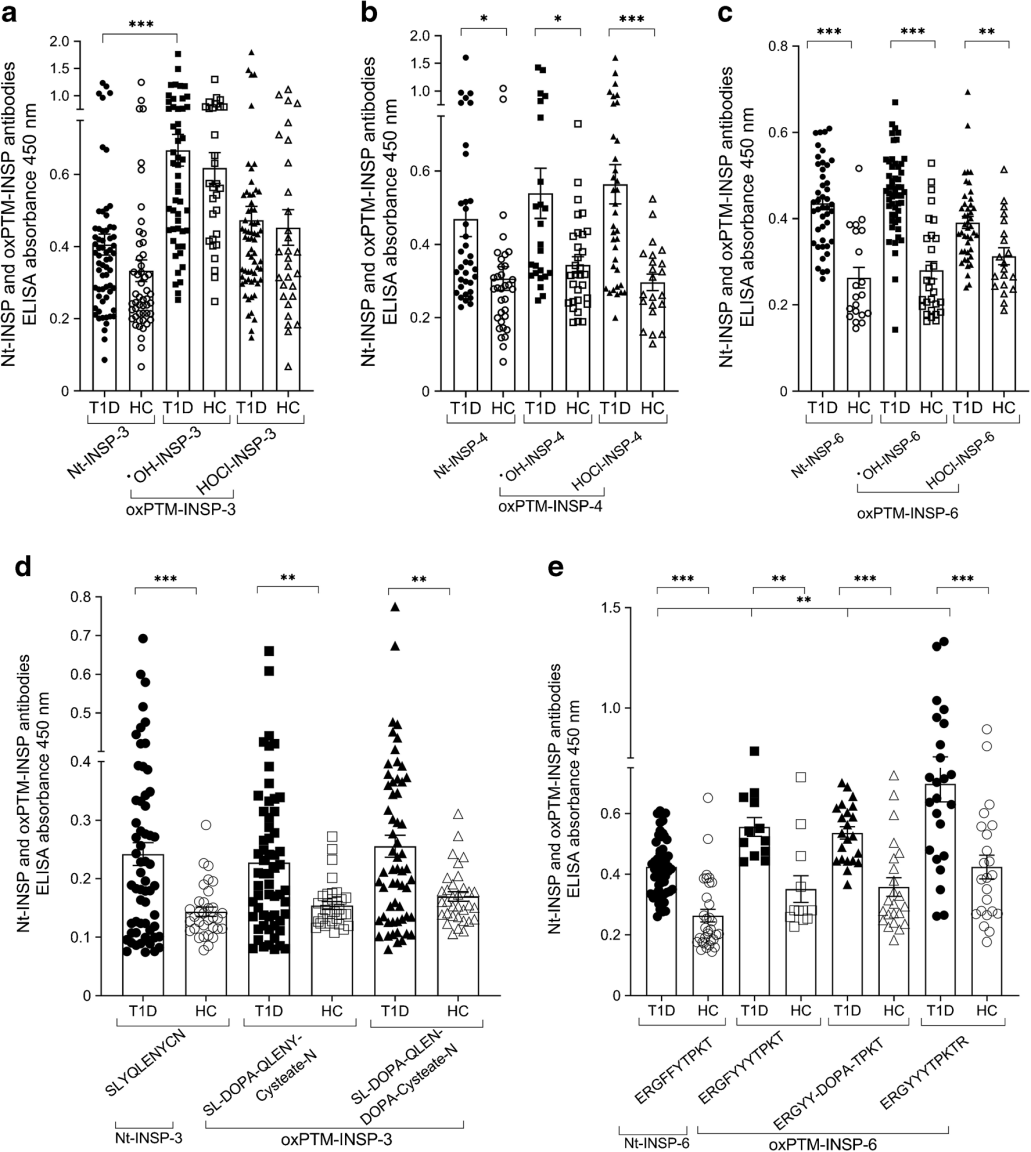
Antibody binding reactivity to neoantigenic INSPs (oxPTM-INSPs) in type 1 diabetes. (**a**–**c**) After oxidation of INSPs by either ^●^OH or HOCl, reactivity of type 1 diabetes serum samples against each of the native or oxPTM-INSP candidates was tested by ELISA. (**a**) oxPTM-INSP-3 (SLYQLENYCN) showed higher reactivity compared with Nt-INSP-3 (*p*<0.001). The highest reactivity for oxPTM-INSP was observed for ^●^OH-modified oxPTM-INSP-3. High background binding in healthy control samples was observed for oxPTM-INSP-3. (**b**) oxPTM-INSP-4 (LVEALYLVCGERGFFYTPKT) and (**c**) oxPTM-INSP-6 (ERGFFYTPKT) displayed significantly greater reactivity in type 1 diabetes samples compared with healthy control samples (*p*=0.0204, *p*=0.0176 and *p*=0.0005 for native, ^●^OH and HOCl oxPTM-INSP-4, respectively; and *p*<0.0001, *p*<0.0001 and *p*=0.0187 for native, ^●^OH and HOCl oxPTM-INSP-6, respectively). (**d**, **e**) In silico-oxidised oxPTM-INSP-3 derivatives of SLYQLENYCN included SL-DOPA-QLENY-Cysteate-N and SL-DOPA-QLEN-DOPA-Cysteate-N (**d**). In silico-oxidised oxPTM-INSP-6 derivatives of ERGFFYTPKT included ERGYYYTPKT, ERGYY-DOPA-TPKT and ERGYYYTPKTR (**e**). (**d**) Binding to SL-DOPA-QLENY-Cysteate-N and SL-DOPA-QLEN-DOPA-Cysteate-N was similar to binding to SLYQLENYCN (*p*=NS). (**e**) In type 1 diabetes patients, binding to ERGYYYTPKT or ERGYYYTPKTR was higher than binding to native ERGFFYTPKT (*p*≤0.008). Similarly, a significant increase in binding to ERGYY-DOPA-TPKT was observed compared with the native ERGFFYTPKT (*p*=0.008). Multiple comparisons were adjusted for using the Holm–Sidak test; *p<0.05; **p<0.01; ***p<0.001. Cut-off points of positivity (binders) in the antibody ELISA for each peptide were defined by the mean absorbance of healthy control samples to the corresponding Nt-INSP plus 3×SEM. HC, healthy control; T1D, type 1 diabetes

Identified peptide candidates were synthesised and exposed to either HOCl or ^●^OH to generate oxPTM-INSPs that were first assessed by time of flight–electrospray ionisation positive mode–total ion chromatogram (TOF-MS/ES+TIC) to confirm modification (ESM Fig. 4). Antibody response against Nt-INSPs and oxPTM-INSPs was evaluated by ELISA using sera from Study cohort 1. Serum antibody binding experiments revealed the highest number of type 1 diabetes binders for ^●^OH-modified oxPTM-INSP-3 (86% binders, mean absorbance=0.667±0.044; cut-off defined as mean binding of healthy control to Nt-INSP-3 plus 3×SEM), HOCl-modified oxPTM-INSP-4 (66% binders, mean absorbance=0.563±0.053; cut-off defined as mean binding of healthy control to Nt-INSP-4 plus 3×SEM) and ^●^OH-modified oxPTM-INSP-6 (83% binders, mean absorbance=0.461±0.013; cut-off defined as mean binding of healthy control to Nt-INSP-6 plus 3×SEM) (Fig. 2a–c, ESM Table 3). No significant reactivity was observed for INSP-1, INSP-2 and INSP-5 (data not shown). For oxPTM-INSP-3, we observed high background binding of healthy control samples (*p*>0.05; Fig. 2a, ESM Table 3). For oxPTM-INSP-4 and oxPTM-INSP-6, binding of type 1 diabetes serum was significantly stronger compared with control samples (*p*=0.0204, *p*=0.0176 and *p*=0.0005 for native, ^●^OH and HOCl oxPTM-INSP-4; and *p*<0.0001, *p*<0.0001 and *p*=0.0187 for native, ^●^OH and HOCl oxPTM-INSP-6; Fig. 2b,c, ESM Table 3). INSP-6 showed the highest specificity and sensitivity, with AUCs of 0.879, 0.875 and 0.740 for native, ^●^OH-modified and HOCl-modified INSP-6 (ESM Table 3, ESM Fig. 5).

### Designing in silico oxPTM-INSPs

We designed in silico multiple oxPTM-INSP derivatives corresponding to one or more aminoacidic modification. For INSP-3 (SLYQLENYCN) we synthesised the following oxPTM-INSP-3 derivatives: SL-DOPA-QLENY-Cysteate-N where tyrosine (Y) was converted to DOPA only in one position and cysteine (C) to Cysteate. An additional oxPTM-INSP-3 was synthesised where both Y residues were converted to DOPA: SL-DOPA-QLEN-DOPA-Cysteate-N. To make the in silico oxPTM-INSP-6 of ERGFFYTPKT, phenylalanine (F) was converted to Y, and Y to DOPA. We thus synthesised two oxPTM-INSP-6 versions of ERGFFYTPKT: ERGYYYTPKT and ERGYY-DOPA-TPKT. We also included another oxPTM-INSP-6 version with a C-terminal arginine (R), ERGYYYTPKTR, as this sequence was seen in several MS profiles and R is the amino acid in the junction with the proinsulin C-peptide (ESM Table 2). Sequences of native peptides and their corresponding in silico oxPTM peptides were confirmed by UPLC-qTOF/MS^e^ (ESM Results, Peptide sequence confirmation by UPLC-qTOF/MS^e^, ESM Fig. 6). Structural changes induced by oxPTM were then studied by circular dichroism analysis (ESM Results, Structural changes in the oxPTM-INSPs compared with native peptides, ESM Fig. 7).

### Antibody reactivity of type 1 diabetes serum against in silico-modified oxPTM-INSPs

In type 1 diabetes patients (Study cohort 1), we observed a non-significant increase in binding to SL-DOPA-QLENY-Cysteate-N and SL-DOPA-QLEN-DOPA-Cysteate-N (54% and 57% binders, respectively) compared with SLYQLENYCN (49% binders, *p*>0.05); binding of type 1 diabetes samples was, however, significantly more frequent compared with healthy control samples (9%, 17% and 30% of control samples bound to SLYQLENYCN, SL-DOPA-QLENY-Cysteate-N and SL-DOPA-QLEN-DOPA-Cysteate-N, with *p*=0.0006, *p*=0.0112 and *p*=0.0029 vs type 1 diabetes, respectively) (Fig. 2d, ESM Table 3). There was no increase in specificity/sensitivity of binding to oxPTM-INSP-3 derivatives compared with the Nt-INSP-3, with AUCs of 0.670, 0.707 and 0.664, for SLYQLENYCN, SL-DOPA-QLENY-Cysteate-N and SL-DOPA-QLEN-DOPA-Cysteate-N, respectively (ESM Table 3, ESM Fig. 5).

We observed a significantly increased binding of type 1 diabetes samples to both ERGYYYTPKT and ERGYYYTPKTR, with 100% and 88% binders, respectively, compared with 25% and 48% binders in control samples, respectively (*p*≤0.004). In type 1 diabetes patients, binding to oxPTM-INSP-6 derivative ERGYYYTPKT or ERGYYYTPKTR was significantly higher compared with the native ERGFFYTPKT (*p*≤0.008). Similarly, a significant increase in binding to ERGYY-DOPA-TPKT was observed, compared with the native ERGFFYTPKT (*p*=0.008; Fig. 2e). We did not observe a significant difference in specificity/sensitivity of Nt-INSP-6 vs in silico-modified oxPTM-INSP-6 derivatives, with AUCs of 0.8686, 0.8542 and 0.8340, respectively (ESM Table 3, ESM Fig. 5).

**Fig. 3:**
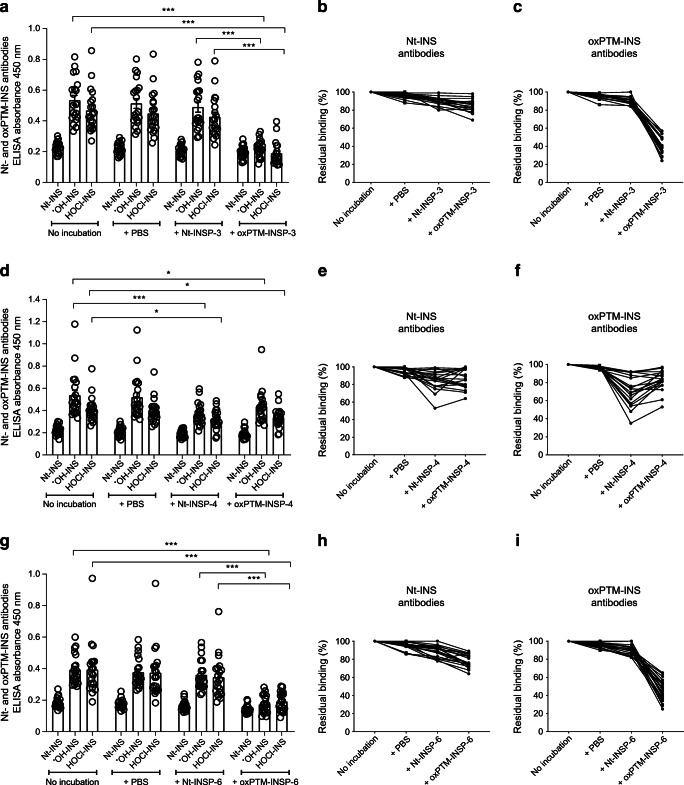
Serum binding specificity to neoantigenic INSPs (oxPTM-INSPs). The figure shows the residual antibody binding to Nt-INS or oxPTM-INS with and without preincubation of type 1 diabetes serum samples with Nt- or oxPTM-INSP-3 (**a**, **b**, **c**), Nt- or oxPTM-INSP-4 (**d**, **e**, **f**), or Nt- or oxPTM-INSP-6 (**g**, **h**, **i**). Preincubation of type 1 diabetes serum samples with oxPTM-INSP-3 and oxPTM-INSP-6, but not with unmodified native peptides, strongly inhibited binding to oxPTM-INS (*p*<0.001), indicating the presence of antigen-binding sites specific to these oxPTM-INSPs. Nt-INSP-4 displayed a comparable inhibition to oxPTM-INSP-4. Percentage residual binding to Nt-INS (**b**, **e**, **h**) and oxPTM-INS antibodies (**c**, **f**, **i**) is shown for each type 1 diabetes sample tested. Antibodies to ^●^OH-INS are used as an example for oxPTM-INS. Each line in the figure panels represents the percentage binding of a serum sample from a single donor with type 1 diabetes to either Nt-INS (**b**, **e**, **h**) or oxPTM-INS (**c**, **f**, **i**) after preincubation with Nt-INSP-3 or oxPTM-INSP-3 (**b**, **c**); Nt-INSP-4 or oxPTM-INSP-4 (**e**, **f**); or Nt-INSP-6 or oxPTM-INSP-6 (**h**, **i**), relative to binding to Nt-INS or oxPTM-INS without peptide competitors (100%). Multiplicity was adjusted for using the Holm–Sidak test; *p<0.05; ***p<0.001

A competitive displacement assay was performed to evaluate serum binding specificities to oxPTM-INSPs by pre-incubating sera with Nt- or oxPTM-INSPs. Interestingly, oxPTM-INSP-3 and oxPTM-INSP-6, but not Nt-INSP-3 or Nt-INSP-6, were able to inhibit the binding of type 1 diabetes samples to oxPTM-INS (*p*<0.001), but not to Nt-INS (Fig. 3a–c,g–i). Competition with combined oxPTM-INSP-3 and oxPTM-INSP-6 did not increase blocking to oxPTM-INS binding compared with a single peptide (data not shown). Nt-INSP-4, however, displayed a comparable inhibition compared with oxPTM-INSP-4 (Fig. 3d–f).

### T cell stimulation with oxPTM-INSPs

To evaluate the immune cell response against the oxPTM-INSPs, we performed CD4^+^ and CD8^+^ T cell proliferation experiments using freshly isolated PBMCs (Study cohort 2, Table 1). Response was calculated as SI over unstimulated T cells.

**Fig. 4:**
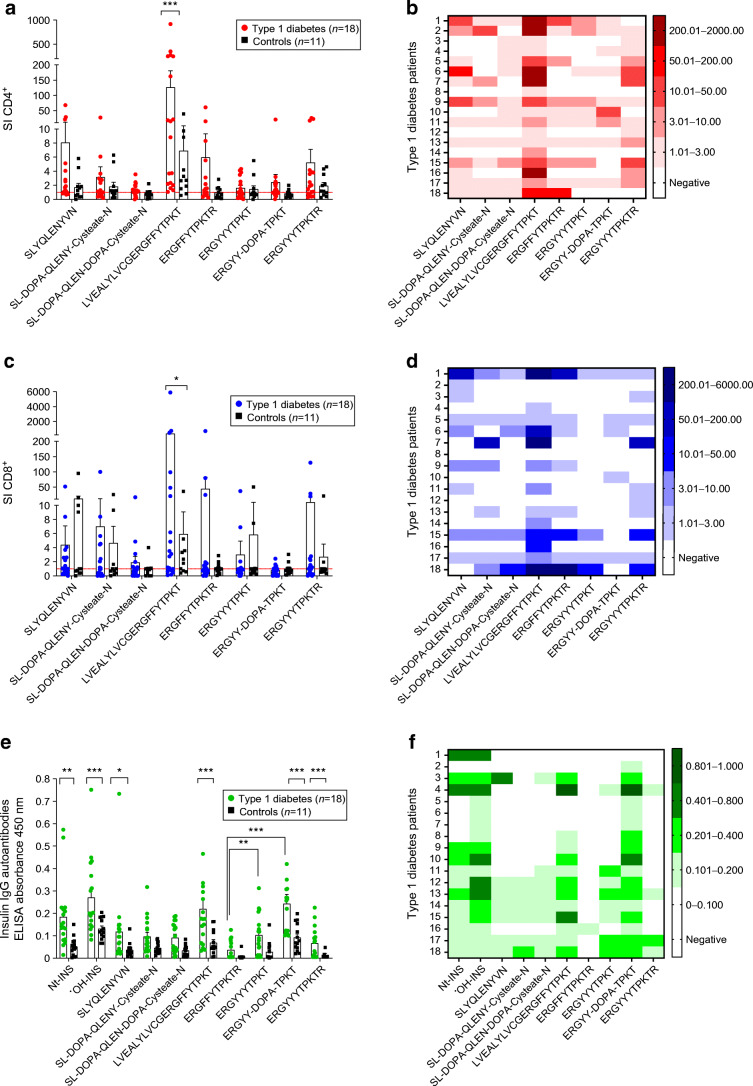
oxPTM-INSPs stimulate T cell and autoantibody responses in type 1 diabetic individuals. The figure shows the CD4^+^ (**a**, **b**, red), CD8^+^ (**c**, **d**, blue) and IgG autoantibody (**e**, **f**, green) responses against Nt-INSPs and oxPTM-INSPs. Nt-INSP-4 (LVEALYLVCGERGFFYTPKT) and different oxidative derivatives of oxPTM-INSP-6 (ERGYYYTPKTR) are the main targets. Heatmaps show the degree of response heterogeneity within type 1 diabetic individuals, with some individuals preferentially responding to different oxPTM-INSP formats derived from the same peptide sequence. (**b**, **d, f**) Heatmaps of reactivity for each individual with type 1 diabetes tested against the various Nt-INS-Ps and oxPTM-INSPs. Multiplicity-adjusted *p* values: *p<0.05; **p<0.01; ***p<0.001

We found that Nt-INSP-4 (LVEALYLVCGERGFFYTPKT) induced the strongest stimulation in type 1 diabetes compared with control samples for both CD4^+^ (mean SI: 119.8±51.69 vs 6.89±3.4, *p*<0.001; Fig. 4a) and CD8^+^ T cells (mean SI: 405.8±325.5 vs 5.948±3.125, *p*=0.049; Fig. 4c). Of note, as highlighted by the heatmaps in Fig. 4b,d, heterogeneous responses also to other peptides were evident across different individuals with type 1 diabetes, with some preferentially responding to various derivatives of oxPTM-INSP-3 (SL-DOPA-QLENY-Cysteate-N, SL-DOPA-QLEN-DOPA-Cysteate-N) and oxPTM-INSP-6 (ERGYYYTPKT, ERGYY-DOPA-TPKT, ERGYYYTPKTR). To better assess specificity of T cell stimulation in type 1 diabetes compared with control participants, we analysed response according to different SI cut-offs. When using an SI>3, we found a larger number of individuals with type 1 diabetes with a CD4^+^ response to oxPTM-INSP-6 derivatives compared with control participants (66.7% vs 27.3%; *p*=0.039), while response to Nt-INSP-4 and oxPTM-INSP-3 was similar between type 1 diabetic and control participants (Nt-INSP-4: 66.7% vs 45.5%; oxPTM-INSP-3: 22.2% vs 9.1%) (ESM Table 4). When comparing response to oxPTM-INSPs and Nt-INSPs among type 1 diabetes patients, we found that CD4^+^ response to oxPTM-INSP-6 was more frequent compared with Nt-INSP-6 (66.7% vs 27.8%; *p*=0.045) (Fig. 4a,b, ESM Table 4). CD8^+^ T cell responses to the tested peptides were also common in individuals with type 1 diabetes, who responded with similar frequency to oxPTM-INSP-6 and Nt-INSP-4 (72.2% of patients showed SI>1 for both); such response was higher in type 1 diabetic compared with control participants for oxPTM-INSP-6 (72.2% vs 27.3%; *p*=0.02), but not for Nt-INSP-4 (72.2% vs 63.6%; *p*=NS) (ESM Table 5). Higher SI cut-offs did not reveal significant differences between groups (ESM Table 5).

**Fig. 5:**
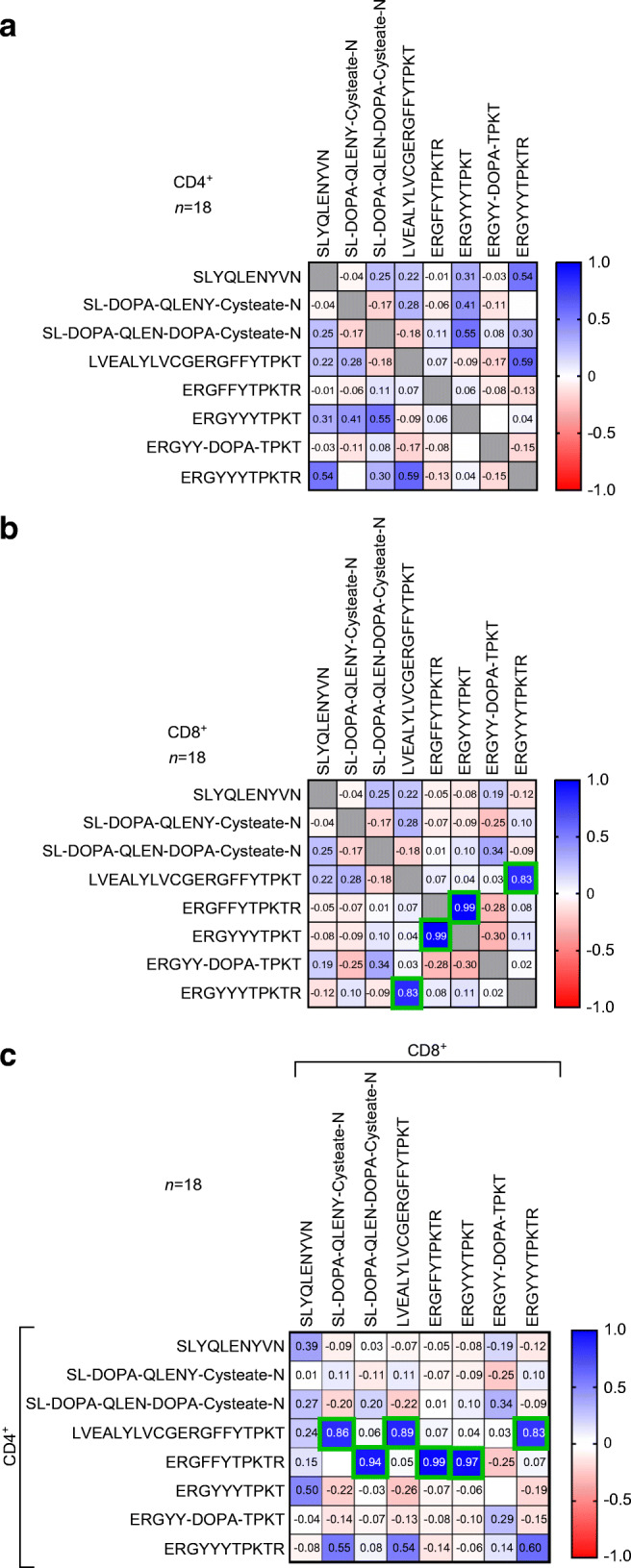
Correlation matrix of CD4^+^ (**a**) and CD8^+^ (**b**) responses to oxPTM-INSPs. T cell stimulation with Nt-INSP-4 (LVEALYLVCGERGFFYTPKT) strongly correlated with stimulation with oxPTM-INSP-6 (ERGYYYTPKTR) containing two aminoacidic oxidations (conversion of Phe^24^ and Phe^25^ to Tyr), for CD8^+^ (*r*=0.83, *p*=0.002) responses. There was no correlation between stimulation by Nt-INSP-4 and stimulation by Nt-INSP-6 (ERGFFYTPKTR) containing the native aminoacidic sequence (**a**, **b**). CD4^+^ stimulatory response to Nt-INSP-4 correlated with CD8^+^ responses to Nt-INSP-4 (*r*=0.89; *p*=0.0017), oxPTM-INSP-6 (*r*=0.83; *p*=0.002) and oxPTM-INSP-3 (SL-DOPA-QLENY-Cysteate-N) (*r*=0.86; *p*=0.0018) (**c**). All analyses are corrected for multiple comparisons, with statistically significant correlations highlighted by green squares

Correlation analysis showed association between T cell responses to the oxPTM-INS-6 derivative ERGYYYTPKTR (but not Nt-INSP-6) and Nt-INSP-4, for both CD4^+^ (*r*=0.59, *p*=0.12; Fig. 5a) and CD8^+^ (*r*=0.83, *p*=0.002; Fig. 5b). The CD4^+^ T cell response to Nt-INSP-4 was also strongly correlated with the CD8^+^ T cell response to oxPTM-INSP derivatives ERGYYYTPKTR and SL-DOPA-QLENY-Cysteate-N (*r*≥0.83, *p*≤0.002), but not their native counterparts (Fig. 5c), suggesting an overlap in CD4^+^ and CD8^+^ T cell responses involving Nt-INSP-4, oxPTM-INSP-3 and oxPTM-INSP-6.

We next utilised surface staining for CD45 receptor type C (CD45RA; the long isoform of CD45 that is expressed on naive T cells) and C-C motif chemokine receptor 7 (CCR7) on CD154^+^CD69^+^ T cells to classify epitope-specific T cells as naive (CD45RA^+^CCR7^+^), central memory (TCM, CD45RA^−^CCR7^+^), effector memory (TEM, CD45RA^−^CCR7^−^) or effector memory cells re-expressing CD45RA (TEMRA, CD45RA^+^CCR7^−^). Across five representative individuals with established type 1 diabetes (Cohort 3, ESM Table 1), we detected TCM, TEM and TEMRA, with naive cells also present. Nt-INS- and oxPTM-INS-specific T cells had a higher percentage of naive cells than the influenza control (44.7% and 41.1%, respectively) but appreciable percentages of TCM and TEM were also present, suggesting that there is an existing pool of memory T cells that recognised these INSPs in individuals with type 1 diabetes (ESM Figs 8, 9).

### Correlation between T cell stimulation and antibody response

Participants evaluated for T cell stimulation were also tested for antibody reactivity to either oxPTM-INSPs or oxidised intact insulin (oxPTM-INS) to assess correlations between humoral and cellular responses (Fig. 4e,f). In Study cohort 2, antibody reactivity to oxPTM-INSP-6 was the highest, as observed in Study cohort 1, with 11/18 (61.1%) binding to at least one oxPTM-INSP-6 derivative (*p*<0.001 oxPTM-INSP-6 vs Nt-INSP-6). Detailed analysis of autoantibody response in this cohort is described in the ESM (ESM Results, Antibody binding to oxPTM-INSPs in Study cohort 2).

**Fig. 6:**
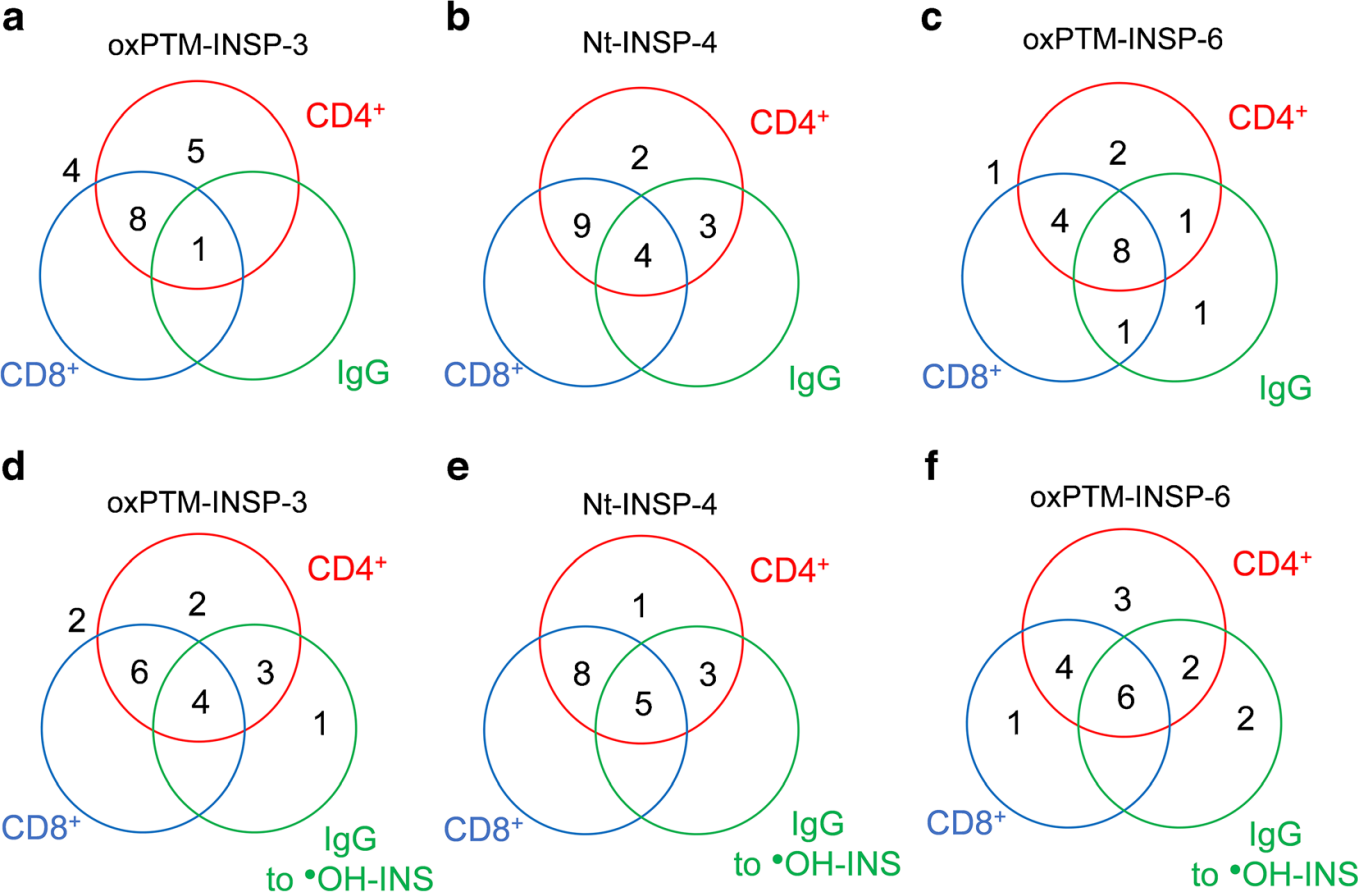
Venn diagrams of CD4^+^, CD8^+^ and antibody responses to oxPTM-INSPs. (**a**, **b**, **c**) Overlap between IgG autoantibody and T cell (CD4^+^ and CD8^+^) responses specific to oxPTM-INSP-3 (**a**), Nt-INSP-4 (**b**) and oxPTM-INSP-6 (**c**). (**d**, **e**, **f**) Overlap between T cell responses to the three INSPs and IgG antibody responses to oxPTM-INS modified by ^●^OH (^●^OH-INS). Overall, 5.5%, 22.2% and 44.4% of patients showed concordant responses involving, simultaneously, CD4^+^, CD8^+^ and IgG towards oxPTM-INSP-3, Nt-INSP-4 and oxPTM-INSP-6, respectively. There is concordance between reactivity to oxPTM-INS and CD4^+^ and CD8^+^ reactivity for all three tested peptides (7/17, 8/18 and 8/18 for oxPTM-INSP-3, Nt-INSP-4 and oxPTM-INSP-6, respectively). An SI>1 was used for definition of a positive T cell response

We then analysed the extent of correlation between CD4^+^, CD8^+^ and IgG antibody responses. CD4^+^ and CD8^+^ responses to oxPTM-INSP-3 overlapped in 9/18 (50.0%), but only 1/18 patients (5.5%) showed concordant antibody reactivity (Fig. 6a). The CD4^+^ T cell response to Nt-INSP-4 frequently overlapped with CD8^+^ (13/18 [72.2%]), and to a lesser extent with antibodies (7/18 [38.8%]). Overall, 4/18 (22.2%) patients had a concordant CD4^+^, CD8^+^ and antibody response to Nt-INSP-4 (Fig. 6b). CD4^+^ response to oxPTM-INSP-6 was linked to both CD8^+^ and/or antibodies: 12/18 (66.7%) patients had concordant CD4^+^ and CD8^+^ responses, while 9/18 (50%) patients had concordant CD4^+^ and antibody responses. Overall, 8/18 (44.4%) patients showed an immune response involving simultaneously CD4^+^, CD8^+^ and antibodies (Fig. 6c). CD4^+^ T cell stimulation with Nt-INSP-4, oxPTM-INSP-6 and oxPTM-INSP-3 was associated with antibody reactivity to oxPTM-INS in 8/18 (44.4%), 8/18 (44.4%) and 7/18 (38.9%) participants with type 1 diabetes. Concordant autoimmune response to oxPTM-INSPs involving simultaneously CD4^+^ and CD8^+^ T cells and autoantibodies to oxPTM-INS was seen in 5/18 (27.8%), 6/18 (33.3%) and 4/18 (22.2%) participants with type 1 diabetes for Nt-INSP-4, oxPTM-INSP-6 and oxPTM-INSP-3, respectively (Fig. 6d–f), suggesting that CD4^+^ T cell response to these peptides is required to generate CD8^+^ and/or antibody responses to oxPTM-INS.

We next performed hierarchical cluster analysis (Euclidean distance, Ward’s method) of patients, and of peptides. Hierarchical cluster analysis and PCA revealed association between responses to different oxPTM-INSPs and identify clustering of type 1 diabetes vs healthy control samples. We observed association between Nt-INSP-4 and oxPTM-INS-P6, ERGYYYTPKTR for CD4^+^ and CD8^+^. For IgG response, ERGYY-DOPA-TPKT is associated with insulin modified by ^●^OH (^●^OH-INS). We also observed clustering of response of type 1 diabetes samples using PCA of all responses, CD4^+^, CD8^+^ and IgG. We observed a cluster of 11 type 1 diabetes samples with principle component 1 (PC1)>0, while the rest clustered with healthy control samples with PC1<0 (ESM Fig. 10).

## Discussion

In this study, we show that neoantigenic INSPs generated by oxPTM are targeted by both circulating autoantibodies and T cells in type 1 diabetes. The main autoimmune response involved three INSPs: B:11–30, B:21–30 and A:12–21, and their respective oxPTM-INSP derivatives: oxPTM-INSP-4 (B:11–30), oxPTM-INSP-6 (B:21–30) and oxPTM-INSP-3 (A:12–21).

We identified multiple cleavage sites after exposure of insulin to oxidants (^●^OH and HOCl). Consistent with literature, cleavage resulting from oxidative damage occurred preferentially between the residues phenylalanine, cysteine, glycine, leucine, valine and tyrosine [24], as well as near the cysteine bridges, especially in the alpha-chain [25]. As previously described [24], we observed structural changes within insulin-derived peptides oxPTM-INSP-3 and oxPTM-INSP-6 as a results of oxidations.

It appears that the nature of the modification itself provides new interaction properties (e.g. additional hydrogen bonding potential), or opens the access to hidden epitopes that could contribute to formation of immunogenic products. Peptide cleavage makes self-antigens more accessible to the immune system and represents a step required for antigen presentation. It has been shown that B:21–29 is a CD8 epitope generated by proteasome cleavage during antigen presentation [24]. Our data suggest that a similar epitope (B:21–30) can result from beta-chain cleavage by oxidation. We speculate that oxidative cleavage may facilitate antigen presentation via a proteasome-independent pathway by providing readily accessible peptides to the immune system.

We found antibodies to oxPTM-INSP-6 in most individuals with type 1 diabetes. Of note, we observed the same pattern of response for oxPTM-INSP-6 that was oxidised in house compared with in silico-designed derivatives (ERGYYYTPKT and ERGYY-DOPA-TPKT), suggesting that oxidation of F to Y and Y to DOPA generates neoepitopes recognised by specific antibodies. Interestingly, methyldopa (an analogue of DOPA) can block the activation of insulin-autoreactive T cells in NOD mice and prevented beta cell loss and IAA in recent-onset type 1 diabetes [26]. In contrast to oxPTM-INSP-6, oxPTM-INSP-3 was less specific, revealing increased background in control participants. This could be due to spontaneous oxidation of cysteine or to presence of a free thiol, which could result in non-specific interaction of SLYQLENYCN, SL-DOPA-QLENY-Cysteate-N and SL-DOPA-QLEN-DOPA-Cysteate-N. Further chemistry studies will need to address this point in future work. Data from oxPTM-INSP-4 did not clearly substantiate the importance of oxPTM for antibody response to INSP-4. Indeed, Nt-INSP-4 blocked type 1 diabetes serum binding to oxPTM-INS like oxPTM-INSP-4. A similar result was observed for the T cells, as stimulation with Nt-INSP-4 was stronger compared with other oxPTM-INSPs.

Antibodies to oxPTM-INSP-6 coincided with cellular responses in most cases, implying that antibody reactivity to oxPTM-INS is dependent on CD4^+^ T cell activation and often associates with CD8^+^ response. Antibody reactivity to oxPTM-INSP-6 (B:21-30) and Nt-INSP-4 (B:11–30) strongly correlated with antibodies to oxPTM-INS. Furthermore, immune responses to oxPTM-INSP-6 and Nt-INSP-4 often coexisted within patients. Sequence homology between the two peptides cannot fully explain the overlap in immune response, because the association was specific to oxPTM-INSP-6 rather than Nt-INSP-6. It is possible that, when modified, oxPTM-INSP-6 gains a structural conformation similar to the C-terminal part of the longer Nt-INSP-4. A second possibility is that the B:11–30 peptide is autoxidised during experimental procedures. We were not able to systematically design/analyse in silico derivatives of INSP-4. Within the INSP-4 sequence LVEALYLVCGERGFFYTPKT, there are at least five oxidatively modifiable amino acid residues corresponding to dozens of potential combinations, in which various numbers of the cysteine, tyrosines and phenylalanines are either oxidised or not at various locations within the peptide. Thus, we had to restrict our in silico-oxidised peptides analysis to the shorter INSP-6 ERGFFYTPKT and INSP-2 LVEALYLVCGER, containing lower numbers of oxidation-susceptible amino acid residues. No reactivity was observed against LVEALYLVCG. Previously, it has been shown that simple exposure to ambient air can induce oxidation of the INSP B:9–23 [27], which is targeted by gamma delta T cells in NOD mice. Intermolecular epitope spreading, involving Nt- and oxPTM-INS and their derived peptides (Nt-INSP-4 and oxPTM-INSP-6), is another potential mechanism. Together, these findings suggest that Nt-INSP-4 and oxPTM-INSP-6 peptides are potential T cell and antibody neoepitopes in type 1 diabetes. We performed a pilot study to identify the T cell subsets stimulated by oxPTM-INSPs, finding an existing pool of memory T cells that recognise oxPTM-INSPs in individuals with type 1 diabetes. Further studies with a larger sample size will be needed to confirm this observation.

Our findings shed light on type 1 diabetes pathogenesis and support the hypothesis that oxidative stress, and hence oxPTM, may have a pathogenic role in the disease. Several of the main putative aetiopathogenic factors linked to the disease have been shown, or potentially are able, to generate oxidative stress [28–30]. For example, numerous virus infections (including by pathogens involved in type 1 diabetes, such as Coxsakieviruses) exert many kinds of oxidative stress in the host [31]. Superoxide production following Coxsakievirus B3 infection may exacerbate beta cell destruction in an experimental model of type 1 diabetes by influencing proinflammatory macrophage responses [31], thus mechanistically linking oxidative stress, inflammation and diabetogenic virus infections. Furthermore, circulating markers of oxidative stress are increased not only in established type 1 diabetes [32], but also in euglycaemic individuals at risk for developing the disease [33]. According to a recent proteomic study, children at risk of diabetes display a consistent pattern of proteins involved in oxidative stress, which was higher before seroconversion to islet autoantibodies. This indicates that oxidative stress exists in the initial stage of type 1 diabetes progression, prior to the earliest sign of islet autoimmunity [34]. Cohort studies have also shown that iron overload is associated with increased risk of type 1 diabetes [35, 36]. Iron salts act as a catalyst in Fenton chemistry by converting, under acidic conditions, hydrogen peroxide to ^●^OH, which is highly oxidising. Finally, diabetes can be induced experimentally in rats by feeding with alloxan or streptozotocin, two substances that work by generating ROS and selective damage to beta cells [37]. Of note, low-dose streptozotocin leads to insulitis and generates an immunological alteration in the islets that, already 30 years ago, has been hypothesised to be the result of a neoantigenic epitope elicited by streptozotocin toxicity [38].

Identification of oxidative neoantigenic peptides of insulin may also have clinical implications for the knowledge of T and B cell specificities to assist in the development of targeted immune tolerance as well as in diagnosis, patient characterisation, and pre- and post-therapy immune monitoring. Our discovery may impact the designing of optimal autoantibody and T cell assays for early detection of processes forgoing or leading to type 1 diabetes.

In conclusion, our findings support the concept that oxidative stress, and neoantigenic epitopes generated by oxPTM of beta cell antigens such as insulin, may be involved in the pathogenesis of type 1 diabetes. Future studies should address the validation of oxPTM-INSPs as disease biomarkers and assess their potential as part of an immune intervention for disease prevention.

## Supplementary Information

ESM (PDF 1531 kb)

## Data Availability

Data can be shared upon request to A. Nissim: a.nissim@qmul.ac.uk.

## Abbreviations

CCR7: C-C motif chemokine receptor type 7
CD45RA: CD45 receptor type C
DOPA: Dihydroxyphenylalanine
GADA: GAD autoantibodies
HOCl: Hypochlorous acid
IA-2A: Tyrosine phosphatase-like molecule IA-2 autoantibodies
IAA: Insulin autoantibodies
IMDIAB: Immunotherapy of Diabetes
INSP: Insulin peptide
Nt-INS: Native insulin
Nt-INSP: Native insulin peptide
^●^OH: Hydroxyl radical
^●^OH-INS: Insulin modified by ^●^OH
oxPTM: Oxidative post-translational modifications
oxPTM-INS: Oxidative post-translationally modified insulin
oxPTM-INSP: Oxidative post-translationally modified insulin peptide
PBMC: Peripheral blood mononuclear cell
PC1: Principal component 1
PCA: Principal component analysis
PTM: Post-translational modifications
SI: Stimulation index
TCM: Central memory T cells
TEM: Effector memory T cells
TEMRA: Effector memory T cells re-expressing CD45RA
UPLC-qTof/MS^e^: Ultra-performance LC coupled with an electrospray ionisation quadrupole time-of-flight mass spectrometer operating in MS exponential mode

## References

[1] Atkinson MA, Eisenbarth GS, Michels AW (2014). Type 1 diabetes. Lancet.

[2] Bluestone JA, Herold K, Eisenbarth G (2010). Genetics, pathogenesis and clinical interventions in type 1 diabetes. Nature.

[3] Bonifacio E (2015). Predicting type 1 diabetes using biomarkers. Diabetes Care.

[4] Ziegler AG, Rewers M, Simell O (2013). Seroconversion to multiple islet autoantibodies and risk of progression to diabetes in children. JAMA.

[5] Babon JA, DeNicola ME, Blodgett DM (2016). Analysis of self-antigen specificity of islet-infiltrating T cells from human donors with type 1 diabetes. Nat Med.

[6] Coppieters KT, Dotta F, Amirian N (2012). Demonstration of islet-autoreactive CD8 T cells in insulitic lesions from recent onset and long-term type 1 diabetes patients. J Exp Med.

[7] Pietropaolo M, Towns R, Eisenbarth GS (2012). Humoral autoimmunity in type 1 diabetes: prediction, significance, and detection of distinct disease subtypes. Cold Spring Harb Perspect Med.

[8] Michels AW, Landry LG, McDaniel KA (2017). Islet-Derived CD4 T Cells Targeting Proinsulin in Human Autoimmune Diabetes. Diabetes.

[9] James EA, Mallone R, Kent SC, DiLorenzo TP (2020). T-Cell Epitopes and Neo-epitopes in Type 1 Diabetes: A Comprehensive Update and Reappraisal. Diabetes.

[10] Laine AP, Holmberg H, Nilsson A (2007). Two insulin gene single nucleotide polymorphisms associated with type 1 diabetes risk in the Finnish and Swedish populations. Dis Markers.

[11] McGinty JW, Chow IT, Greenbaum C, Odegard J, Kwok WW, James EA (2014). Recognition of posttranslationally modified GAD65 epitopes in subjects with type 1 diabetes. Diabetes.

[12] Rondas D, Crevecoeur I, D'Hertog W (2015). Citrullinated glucose-regulated protein 78 is an autoantigen in type 1 diabetes. Diabetes.

[13] Arribas-Layton D, Guyer P, Delong T (2020). Hybrid Insulin Peptides Are Recognized by Human T Cells in the Context of DRB1*04:01. Diabetes.

[14] Delong T, Wiles TA, Baker RL (2016). Pathogenic CD4 T cells in type 1 diabetes recognize epitopes formed by peptide fusion. Science.

[15] Yang C, Kelaini S, Caines R, Margariti A (2018). RBPs Play Important Roles in Vascular Endothelial Dysfunction Under Diabetic Conditions. Front Physiol.

[16] Mannering SI, Harrison LC, Williamson NA (2005). The insulin A-chain epitope recognized by human T cells is posttranslationally modified. J Exp Med.

[17] Acevedo-Calado M, James EA, Morran MP (2017). Identification of Unique Antigenic Determinants in the Amino Terminus of IA-2 (ICA512) in Childhood and Adult Autoimmune Diabetes: New Biomarker Development. Diabetes Care.

[18] Strollo R, Vinci C, Arshad MH (2015). Antibodies to post-translationally modified insulin in type 1 diabetes. Diabetologia.

[19] Strollo R, Vinci C, Napoli N, Pozzilli P, Ludvigsson J, Nissim A (2017). Antibodies to post-translationally modified insulin as a novel biomarker for prediction of type 1 diabetes in children. Diabetologia.

[20] Strollo R, Vinci C, Napoli N (2019). Antibodies to oxidized insulin improve prediction of type 1 diabetes in children with positive standard islet autoantibodies. Diabetes Metab Res Rev.

[21] Micsonai A, Wien F, Kernya L (2015). Accurate secondary structure prediction and fold recognition for circular dichroism spectroscopy. Proc Natl Acad Sci U S A.

[22] Yang J, Wen X, Xu H (2017). Antigen-Specific T Cell Analysis Reveals That Active Immune Responses to beta Cell Antigens Are Focused on a Unique Set of Epitopes. J Immunol.

[23] Delong ER, Delong DM, Clarkepearson DI (1988). Comparing the Areas under 2 or More Correlated Receiver Operating Characteristic Curves - a Nonparametric Approach. Biometrics.

[24] Torosantucci R, Mozziconacci O, Sharov V, Schoneich C, Jiskoot W (2012). Chemical modifications in aggregates of recombinant human insulin induced by metal-catalyzed oxidation: covalent cross-linking via michael addition to tyrosine oxidation products. Pharm Res.

[25] Guedes S, Vitorino R, Domingues MR, Amado F, Domingues P (2010). Oxidative modifications in glycated insulin. Anal Bioanal Chem.

[26] Ostrov DA, Alkanani A, McDaniel KA (2018). Methyldopa blocks MHC class II binding to disease-specific antigens in autoimmune diabetes. J Clin Invest.

[27] Aydintug MK, Zhang L, Wang C (2014). gammadelta T cells recognize the insulin B:9–23 peptide antigen when it is dimerized through thiol oxidation. Mol Immunol.

[28] Cnop M, Welsh N, Jonas JC, Jorns A, Lenzen S, Eizirik DL (2005). Mechanisms of pancreatic beta-cell death in type 1 and type 2 diabetes - Many differences, few similarities. Diabetes.

[29] Hanson C, Lyden E, Furtado J, Van Ormer M, Anderson-Berry A (2016). A Comparison of Nutritional Antioxidant Content in Breast Milk, Donor Milk, and Infant Formulas. Nutrients.

[30] Sharma V, Kalim S, Srivastava MK, Nanda S, Mishra S (2009). Oxidative stress and coxsackievirus infections as mediators of beta cell damage: A review. Sci Res Essays.

[31] Burg AR, Das S, Padgett LE, Koenig ZE, Tse HM (2018). Superoxide Production by NADPH Oxidase Intensifies Macrophage Antiviral Responses during Diabetogenic Coxsackievirus Infection. J Immunol.

[32] Marra G, Cotroneo P, Pitocco D (2002). Early increase of oxidative stress and reduced antioxidant defenses in patients with uncomplicated type 1 diabetes: a case for gender difference. Diabetes Care.

[33] Matteucci E, Giampietro O (2000). Oxidative stress in families of type 1 diabetic patients. Diabetes Care.

[34] Liu CW, Bramer L, Webb-Robertson BJ, Waugh K, Rewers MJ, Zhang Q (2018). Temporal expression profiling of plasma proteins reveals oxidative stress in early stages of Type 1 Diabetes progression. J Proteome.

[35] Ellervik C, Mandrup-Poulsen T, Andersen HU (2011). Elevated transferrin saturation and risk of diabetes: three population-based studies. Diabetes Care.

[36] Ellervik C, Mandrup-Poulsen T, Nordestgaard BG (2001). Prevalence of hereditary haemochromatosis in late-onset type 1 diabetes mellitus: a retrospective study. Lancet.

[37] Lenzen S (2008). The mechanisms of alloxan- and streptozotocin-induced diabetes. Diabetologia.

[38] Weide LG, Lacy PE (1991). Low-dose streptozocin-induced autoimmune diabetes in islet transplantation model. Diabetes.

