# Supplementary material for: Autoantibody and T cell responses to oxidative post-translationally modified insulin neoantigenic peptides in type 1 diabetes
Source: Diabetologia. 2022 Oct 7;66(1):132–46. doi: 10.1007/s00125-022-05812-4 (PMC9729141; doi:10.1007/s00125-022-05812-4)
Supplement: Supplementary file 1 — (PDF 1531 kb) [file 125_2022_5812_MOESM1_ESM.pdf]

Electronic supplementary material (ESM)

ESM methods

ELISA assay for antibody detection. The ELISA analysis of native (Nt-INS) and modified insulin (oxPTM-INS) was performed as previously described [18-19]. Briefly, the ELISA plate was coated overnight at 4°C with 100µl per well of modified or native insulin in 0.05 M carbonate/bicarbonate buffer pH 9.6 at 10 µg/ml. The next day, the plates were washed 3 times with 0.1% Tween PBS, followed by blocking for 2 hours at room temperature with 200µl per well of 5% BSA in 0.1% Tween PBS. After washing, 100 µl of 1:200-diluted serum samples in 5% BSA-0.1% Tween PBS were added for additional 2 hours incubation. ELISA plates were then washed 3 times with 0.1% Tween PBS followed by probing with anti-human IgG horse radish peroxidase conjugated (HRP, Sigma) at 1:1,000 dilution in 5% BSA-0.1% Tween PBS for another 1.5 hours incubation. The ELISA plates were washed, and 100µg/ml TMB substrate in 0.1M sodium acetate pH 6.0 plus 2µl/10ml of H_2O_2, were added. Subsequently, the reaction was stopped with 20% v/v sulphuric acid. The optical density (O.D.) was measured at 450 nm using a GENios plate reader and Magellan software (TECAN, Dorset, UK).

Binding of serum to for oxPTM-INS fragments was also done by ELISA. Fraction from the AKTA runs were collected further to 1:10 dilution in PBS before coating microtiter plates for further overnight incubation at 37^{0}C which followed the assay as above.

To assess the binding specificity of serum samples to identified peptides, a competitive ELISA was performed. The ELISA was carried out following the method described above, except that the serum samples were pre-incubated for 2h with and without 10 µg/ml peptides as competitors, before adding the serum samples to the insulin coated ELISA plate.

Peptides synthesis-modification-assessment. The peptides were synthesized using standard Fmoc solid phase chemistry (Shepherd and Atherton). Peptides were cleaved from the appropriate resin using TFA containing silanes and ethane dithiol, which removed all side chain protecting groups. If

the acetonide protecting group had not been fully cleaved using this methodology the peptide was dissolved in an aqueous buffer containing 0.1% TFA and the peptide monitored by MS until complete removal of the group was verified. The peptides were then purified using HPLC on a C18 column (Phenomenex) using a gradient elution profile of water:acetonitrile and 0.1% TFA. Peptides containing cysteate were synthesised using Fmoc-L-Cysteic acid (Carbosynth) and those containing dihydroxyphenylalanine (DOPA) were synthesised using Fmoc-DOPA (acetonide)-OH (Carbosynth). These two amino acids were treated the same as standard Fmoc amino acids.

Peptide Fractionation and Mass Spectrometry. Ultra-performance liquid chromatography coupled with an electrospray ionization quadrupole time-of flight mass spectrometry operating in MSE mode (UPLC-qTof/MS^e) was used to identify all peptides, and to generate fragment ions upon collision induced dissociation (CID) to positively confirm their sequences. The analysis was executed on an ACQUITY H-Class UPLC system (Waters, Milford, USA) coupled to a qTOF High Definition Mass Spectrometer (HDMS) Synapt G2Si, equipped with an electrospray ionisation (ESI) interface (Waters, Milford, USA). All six peptide samples were separated chromatographically using a Waters Acquity UPLC BEH C18 column (1.7 µm, 2.1 mm × 50 mm). The mobile phases consisted of A (LCMS grade Water, 0.1% formic acid) and B (LCMS grade Acetonitrile, 0.1% formic acid), with the following gradient: 0–2 min, 5% B; 2.0–3.0 min, 5%−45% B; 3.0–3.1 min, 45%−90% B; 3.1–4.0 min, 90% B; 4.0–4.1 min, 90%–5% B, 4.1%−5% B. The flow rate was set at 0.45 mL/min. The temperature in the auto sampler and in the column oven was set at 10°C and 60°C, respectively. MS data were collected from m/z 100–1500 Da in positive MSE continuum mode. The electrospray ionization conditions were set as follows: capillary voltage, 3.5 kV; cone voltage, 40 V; cone gas flow, 50 L/h; source temperature, 120°C; desolvation gas flow, 800 L/h; and desolvation temperature, 450°C. Collision energy was set at 4 V in low energy acquisition; whereas high energy collision energy ramp wat set at 10–40 V. Leucine Encephalin (m/z 556.2771) was used for lock mass at a concentration of 200 pg/mL and a flow rate of 20 µl/min. Data acquisition was carried out with

Masslynx v4.2 (Waters, Milford, USA), whereas data were processed using the UNIFI Scientific Information System v1.8 software (Waters, Milford, USA).

Structural changes analysis by Circular dichroism. Structural changes were determined by Circular dichroism. Nt-INSP-6 and oxPTM-INSP-6 were resuspended in PBS buffer while Nt-INSP-3 was resuspended in 50% (v/v) methanol, 50% (v/v) PBS buffer, oxPTM-INSP-3 Ac-SL-DOPA-QLENY-Cysteate-N peptide was resuspended in 25% (v/v) methanol, 75% (v/v) PBS buffer and oxPTM-INSP-3 Ac-SL-DOPA-QLEN-DOPA-Cysteate-N peptide was resuspended in 15% (v/v) methanol, 85% (v/v) PBS buffer. Far-UV CD spectra were measured in a Chirascan (Applied Photophysics) spectropolarimeter thermostated at 20°C. The spectra of peptides (0.05 mg/ml) were recorded from 260 to 200nm, at 0.5nm intervals, 1 nm bandwidth, and a scan speed of 10nm/min. Three accumulations were averaged for each spectrum. Data analysis was carried out using the BeStSel server [21].

Assay for detection of peptide specific T cells. An activation induced marker (AIM) to label and characterize native (Nt-INS) and modified insulin (oxPTM-INS) responsive T cells and influenza specific T cells (as a positive control) was performed as previously described [22]. Briefly, PBMC from subjects with type 1 diabetes were plated at a density of 10 million cells per ml in 24 well plates and pulsed with solvent only (Mock) or specific peptides of interest (oxPTM-INSP, Nt-INSP, or an influenza peptide) in individual wells for 10-14 hours in the presence of an anti-CD40 blocking antibody (Miltenyi Biotec). Activated T cells were labeled with anti-CD154 APC (Miltenyi Biotec) followed by anti-APC beads (Miltenyi Biotec) and magnetically enriched using columns (Miltenyi Biotec), reserving a 1% fraction of the non-enriched cells to determine the total number of T cells in the sample. The enriched and reserved cells were labeled with anti-CD4 BUV395 (BD Biosciences), anti-CD69 PE-Cy7 (BioLegend), anti-CD45RA AF700 (BD Biosciences), anti-CCR7 APC-Cy7 (BioLegend), plus anti-CD14 PerCP-Cy5.5 (BD Biosciences) anti-CD19 PerCP-Cy5.5 (BD Biosciences) and Viaprobe (BD Biosciences) as cell exclusion markers acquired using an BD LSR II cytometer and analyzed using FlowJo software (Tree Star).

ESM results

Peptide sequence confirmation by UPLC-qTOF/MS^{e}. ESM Fig. 6 shows the fragmentation spectra of the three versions of synthetically oxidative modified peptides 3 (oxPTM-INSP-3) and 6 (oxPTM-INSP-6). Native and oxPTM-INSP-3 correspond to common sequence backbone of SLYQLENYCN, [R-NH3]$^{+}$, m/z 1288.551); Ac-SL-DOPA-QLENY-cysteate-N, [R-NH3]$^{+}$, m/z 1352.5162) and Ac-SL-DOPA-QLEN-DOPA-cysteate-N, [R-NH3]$^{+}$, m/z 1368.5091). Fragmentation pattern of oxPTM-INSP-6 versions, ERGFFYTPKT-NH2, [R-NH_3]$^{+}$, m/z 1286.6626; ERGYYYTPKT, [R-NH3]+, m/z 1277.6253 and ERGYY-DOPA-TPKT, [R-NH3]+, m/z 1293.6213 is also shown. Table in ESM Fig. 6 highlights the fragment ions identified with different m/z values as a result of modifications. Detailed CID spectra with 67%-83% matched first generation primary ions within a filtered upper limit of error of measurement of 30 ppm of peptide y and b assigned fragment ions positively identified against their respective putative sequences and expected modifications was collected (not shown).

Structural changes in the oxPTM-INSPs compared to native peptides. Within the context of intact insulin, residues within native peptide 3 form a helix (ESM Fig. 7a). However, the free peptide is significantly unstructured (52%) but with sheet forming propensity (27%) and minor helical conformations (6%). After oxPTM modification, the SL-DOPA-QLENY-cysteate-N peptide became more structured with increased helicity (16%). However, the SL-DOPA-QLEN-DOPA-cysteate-N peptide was more disordered (60%) with complete loss of helical structure. Residues corresponding to native peptide 6 are extended within intact insulin and the free peptide (ERGFFYTPKT) showed both coil (54%) and sheet (29%) conformations. After oxPTM modification ERGYYYTPKT, ERGYY-DOPA-TPKT and ERGYYYTPKTR became significantly more sheet-like in structure (10 to 14% increase), while addition of arginine at the C-terminus in peptide Ac-ERGFFYTPKTR had no effect.

Antibody binding to oxPTM-INSPs in Study cohort 2. When analysing the IgG autoantibody reactivity in Study cohort 2, we found increased binding to NT-INS, $^{\bullet}$OH-INS, and insulin peptides compared to healthy controls ($p<0.001$), with 6/18 (33.3%), 9/18 (50%), and 11/18 (61.1%) type 1 diabetes binders to Nt-INS, $^{\bullet}$OH-INS, and insulin peptides, respectively. Of note, 11/18 (61.1%) bound to at least one oxPTM-INSP-6 derivatives, with ERGYY-Dopa-TPKT being the most reactive peptide (10/18 [55.5%]), while none bound to the Nt-INSP-6 ($p<0.001$); among type 1 diabetes binders to oxPTM-INSP-6, two patients also showed reactivity to Nt-INSP-3 and oxPTM-INSP-3 (SL-Dopa-QLENY-Cysteate-N), respectively, while 7/18 (38.9%) type 1 diabetes patients showed reactivity to the Nt-INSP-4 peptide. Reactivity to the oxPTM-INSP-6 derivative ERGYY-dopa-TPKT, but not the Nt-INSP-6, displayed a positive correlation with Nt-INSP-4 (LVEALYLVCGERGFFYTPKT) ($r=0.92$; $p<0.001$). Antibody reactivity to both ERGYY-DOPA-TPKT and LVEALYLVCGERGFFYTPKT correlated with $^{\bullet}$OH-INS ($r=0.75$, $p<0.001$; and $r=0.76$, $p<0.001$). By contrast, neither Nt-INSP-6 or Nt-INSP-3 correlated with $^{\bullet}$OH-INS antibody reactivity.

ESM Table 1 - Clinical features of subjects with established type 1 diabetes (T1D) from the Benaroya Research Institute.

Patient	Age at Draw (years)	Gender	Disease duration (years)
T1D #1	29	male	10.0
T1D #2	37	male	7.1
T1D #3	39	male	2.1
T1D #4	33	male	8.0
T1D #5	32	male	7.1

ESM Table 2 – Fractions corresponding to small insulin fragments from several AKTA runs that showed reactivity in ELISA were analysed by LC-MS/MS. The analysis was based on the cleaved peptides following the oxidation producing singly charged ions, which are not ordinarily selected for MS/MS in a typical proteomic experiment.

Observed	Mr(expt)	Mr(calc)	Score	Peptide	Peptide #
391.1789	780.3432	780.3436	28	Y.LVCGERG.F + Trioxidation (C)	2
408.1710	814.3275	814.3280	34	L.VCGERGF.F + Trioxidation (C)	
413.2265	824.4384	824.4392	(36)	C.GSHLVEAL.Y	4
413.2269	824.4392	824.4392	38	C.GSHLVEAL.Y	4
452.2342	902.4539	902.4538	30	G.FFYTPKT.R	6
464.7131	927.4116	927.4120	39	Y.LVCGERGF.F + Trioxidation (C)	1
466.1765	930.3384	930.3389	22	Y.QLENYCN.- + Trioxidation (C)	3
466.1769	930.3393	930.3389	(21)	Y.QLENYCN.- + Trioxidation (C)	3
472.7105	943.4065	943.4069	33	L.YLVCGERG.F + Trioxidation (C)	1
480.7449	959.4753	959.4753	44	R.GFFYTPKT.R	6
547.7083	1093.4019	1093.4022	25	L.YQLENYCN.- + Trioxidation (C)	3
564.7703	1127.5261	1127.5281	28	E.ALYLVCGERG.F + Trioxidation (C)	4
600.7817	1199.5488	1199.5492	47	V.EALYLVCGER.G + Trioxidation (C)	4
629.2922	1256.5699	1256.5707	55	V.EALYLVCGERG.F + Trioxidation (C)	4
647.7662	1293.5178	1293.5183	30	C.SLYQLENYCN.- + Trioxidation (C)	3
650.3163	1298.6180	1298.6176	55	L.VEALYLVCGER.G + Trioxidation (C)	4
434.8870	1301.6393	1301.6404	21	C.GERGFFYTPKT.R	1+6
678.8259	1355.6373	1355.6391	49	L.VEALYLVCGERG.F + Trioxidation (C)	1
776.8563	1551.6981	1551.7028	31	L.VCGERGFFYTPKT.R + Trioxidation (C)	1+6
833.3997	1664.7849	1664.7868	37	Y.LVCGERGFFYTPKT.R + Trioxidation (C)	1+6
421.7581	841.5016	841.5022	25	R.VATVSLPR.S	NA
523.2856	1044.5566	1044.5564	58	K.LSSPATLNSR.V	NA
380.2342	758.4538	758.4538	22	D.SIIAEVK.A	NA
416.7476	831.4807	831.4814	30	K.SISISVAR.G	NA
452.2341	902.4536	902.4538	20	G.FFYTPKT.R	6
480.7448	959.4750	959.4753	41	R.GFFYTPKT.R	6
547.7083	1093.4019	1093.4022	20	L.YQLENYCN.- + Trioxidation (C)	3
564.7703	1127.5261	1127.5281	28	E.ALYLVCGERG.F + Trioxidation (C)	2+4
600.7817	1199.5488	1199.5492	47	V.EALYLVCGER.G + Trioxidation (C)	2+4
629.2922	1256.5699	1256.5707	55	V.EALYLVCGERG.F + Trioxidation (C)	2+4+6
647.7662	1293.5178	1293.5183	30	C.SLYQLENYCN.- + Trioxidation (C)	3
650.3163	1298.6180	1298.6176	55	L.VEALYLVCGER.G + Trioxidation (C)	2+4
434.8870	1301.6393	1301.6404	21	C.GERGFFYTPKT.R	6
678.8259	1355.6373	1355.6391	49	L.VEALYLVCGERG.F + Trioxidation (C)	2+4+6
422.7200	843.4254	843.4239	20	A.AFVNQHL.C + Oxidation (HW)	5

ESM Table 3 – Statistical analysis of binding of the various native and oxPTM- insulin peptides (INSP). The most significant binding observed is for insulin peptide 6 (INSP-6) that demonstrate the highest specificity with ROC-AUC >0.8 and 100% binding to the *in silico* modified oxPTM-INSP-6 (ERGYYYTPKT and ERGYY-DOPA-TPKT). Cut-off points of positivity (binders) for each peptide were defined by the mean absorbance of healthy controls to the corresponding Nt-INSP plus three times the standard error of the mean (SEM). All analyses were corrected for multiple comparisons using Holm Sidak's test.

		Type 1 diabetes		Healthy controls		AUC	*p* value
		Mean±SEM	% binders	Mean±SEM	% binders		
***In house* modifications**							
**INSP-3**	Native	0.405±0.029	42	0.333±0.030	19	0.648	0.4744
	oxPTM-•OH	0.667±0.044	86	0.617±0.042	86	0.515	0.6651
	oxPTM-HOCl	0.473±0.037	53	0.452±0.048	40	0.543	0.7259
**INSP-4**	Native	0.469±0.047	50	0.306±0.032	20	0.730	0.0204
	oxPTM-•OH	0.539±0.068	58	0.344±0.022	27	0.705	0.0176
	oxPTM-HOCl	0.563±0.053	66	0.296±0.022	27	0.818	0.0005
**INSP-6**	Native	0.435±0.014	66	0.263±0.023	10	0.879	<0.0001
	oxPTM-•OH	0.461±0.013	83	0.280±0.020	12	0.875	<0.0001
	oxPTM-HOCl	0.391±0.013	43	0.312±0.019	25	0.740	0.0187
***In silico* modifications**							
**INSP-3**	SLYQLENYCN	0.242±0.019	49	0.143±0.007	9	0.670	0.0006
	SL-DOPA-QLENY-Cysteate-N	0.228±0.016	54	0.154±0.006	17	0.707	0.0112
	SL-DOPA-QLEN-DOPA-Cysteate-N	0.256±0.018	57	0.169±0.008	30	0.664	0.0029
**INSP-6**	ERGFFYTPKT	0.423±0.012	63	0.263±0.020	16	0.868	<0.0001
	ERGYYYTPKT	0.556±0.031	100	0.351±0.044	25	0.854	0.004
	ERGYY-DOPA-TPKT	0.536±0.021	100	0.357±0.030	30	0.834	0.0006
	ERGYYYTPKTR	0.697±0.058	88	0.423±0.039	48	0.797	<0.0001

ESM Table 4 – CD4$^+$ T-cell response in subjects with type 1 diabetes and controls according to different stimulatory index (SI) cut-offs.

	Type 1 diabetes (N=18)					Controls (N=11)			
	SI>1	SI>3	SI>5	SI>10		SI>1	SI>3	SI>5	SI>10
Nt-INSP-3									
SLYQLENYVN	13 (72.2%)	5 **(27.8%)**	4 (21.1%)	3 (16.7%)		6 (54.5%)	2 (18.2%)	1 (9.1%)	0 (0.0%)
oxPTM-INSP-3 derivatives									
SL-Dopa-QLENY-Cysteate-N	9 (50.0%)	3 (16.7%)	2 (10.5%)	1 (5.6%)		6 (54.5%)	2 (18.2%)	2 (18.2%)	0 (0.0%)
SL-Dopa-QLEN-Dopa-Cysteate-N	12 (66.7%)	1 (5.6%)	0 (0.0%)	0 (0.0%)		3 (27.3%)	0 (0.0%)	0 (0.0%)	0 (0.0%)
at least one oxPTM-INSP-3 derivative	15 (83.3%)	**4 (22.2%)**	4 (21.1%)	1 (5.6%)		8 (72.2%)	**1 (9.1%)**	2 (18.2%)	0 (0.0%)
Nt-INSP-4									
LVEALYLVCGERGFFYTPKT	18 (100%)	**12 (66.7%)**	10 (5.3%)	9 (50.0%)		9 (81.8%)	**5 (45.5%)**	3 (27.3%)	1 (9.1%)
Nt-INSP-6									
ERGFFYTPKTR	10 (55.6%)	**5 (27.8%)**	4 (21.1%)	2 (11.1%)		4 (36.4%)	0 (0.0%)	0 (0.0%)	0 (0.0%)
oxPTM-INS-6 derivatives									
ERGYY-Dopa-TPKT	9 (50.0%)	1 (5.6%)	1 (5.2%)	1 (5.6%)		3 (27.3%)	0 (0.0%)	0 (0.0%)	0 (0.0%)
ERGYYYTPKT	9 (50.0%)	4 (22.2%)	0 (0.0%)	0 (0.0%)		4 (36.4%)	2 (18.2%)	1 (9.1%)	0 (0.0%)
ERGYYYTPKTR	13 (72.2%)	8 (38.9%)	3 (15.8%)	3 (16.7%)		7 (63.6%)	3 (27.3%)	0 (0.0%)	0 (0.0%)
at least one oxPTM-INSP-6 derivative	15 (83.3%)	**12 (66.7%)**	4 (21.1%)	4 (22.2%)		7 (63.6%)	**3 (27.3%)**	1 (9.1%)	0 (0.0%)

ESM Table 5 – CD8$^+$ T-cell response in subjects with type 1 diabetes and controls according to different stimulatory index (SI) cut-offs.

	Type 1 diabetes (N=18)					Controls (N=11)			
	SI>1	SI>3	SI>5	SI>10		SI>1	SI>3	SI>5	SI>10
Nt-INSP-3									
SLYQLENYVN	9 (50.0%)	4 (22.2%)	2 (11.1%)	1 (5.6%)		4 (36.4%)	4 (36.4%)	4 (36.4%)	2 (18.2%)
oxPTM-INSP-3 derivatives									
SL-Dopa-QLENY-Cysteate-N	8 (44.4%)	5 (27.8%)	3 (16.7%)	1 (5.6%)		4 (36.4%)	3 (27.3%)	3 (27.3%)	2 (18.2%)
SL-Dopa-QLENDopa-Cysteate-N	6 (33.3%)	3 (16.7%)	1 (5.5%)	1 (5.6%)		2 (18.2%)	1 (9.10%)	0 (0.0%)	0 (0.0%)
at least one oxPTM-INSP-3 derivative	10 (55.6%)	6 (33.3%)	2 (10.5%)	2 (10.5%)		5 (45.5%)	3 (27.3%)	3 (27.3%)	2 (18.2%)
Nt-INSP-4									
LVEALYLVCGERGFFYTPKT	**13 (72.2%)**	10 (55.6%)	7 (38.9%)	6 (33.3%)		**7 (63.6%)**	5 (45.5%)	3 (27.3%)	2 (18.2%)
Nt-INSP-6									
ERGFFYTPKTR	8 (44.4%)	3 (16.7%)	3 (16.7%)	3 (16.7%)		3 (27.3%)	0 (0.0%)	0 (0.0%)	0 (0.0%)
oxPTM-INS-6 derivatives									
ERGYY-Dopa-TPKT	5 (27.8%)	0 (0.0%)	0 (0.0%)	0 (0.0%)		2 (18.2%)	1 (9.10%)	0 (0.0%)	0 (0.0%)
ERGYYYTPKT	4 (22.2%)	2 (11.1%)	2 (11.1%)	1 (5.6%)		3 (27.3%)	2 (18.2%)	2 (18.2%)	1(9.1%)
ERGYYYTPKTR	10 (55.6%)	3 (16.7%)	3 (16.7%)	3 (16.7%)		2 (18.2%)	1 (9.10%)	1 (9.1%)	1(9.1%)
at least one oxPTM-INS-6 derivative	**13 (72.2%)***	3 (16.7%)	3 (16.7%)	3 (16.7%)		**3 (27.3%)**	2 (18.2%)	2 (18.2%)	0 (0.0%)

**p* vs. controls = 0.02

ESM Figures

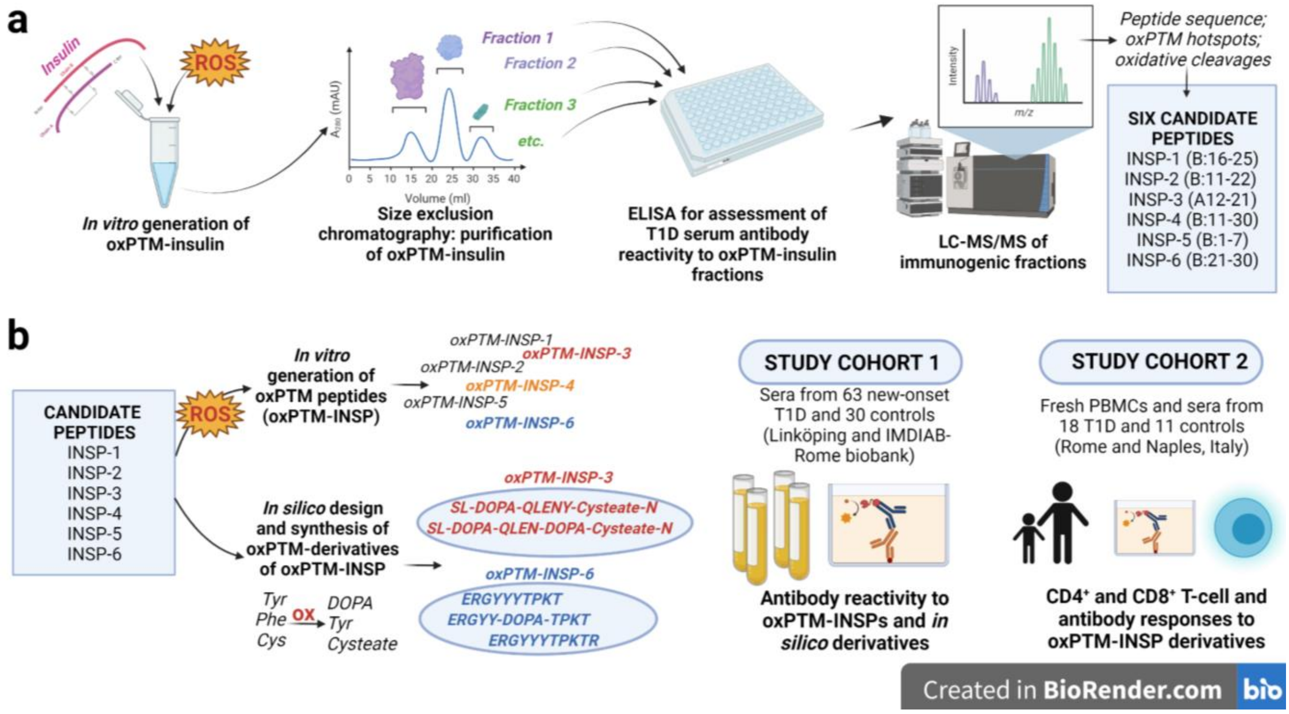

ESM Figure 1. Epitope mapping strategy. a Oxidative posttranslational modifications (oxPTM) of insulin were generated using various reactive oxidants (ROS). Peptide fractions of oxPTM-insulin (oxPTM-INS) were separated by size exclusion chromatography, and immunogenic fractions further characterized by LC/MS-MS. **b**. The identified peptide candidates were then oxidized *in house* (oxPTM-INS peptides), or *in silico* modified to generate multiple oxPTM-INS peptides derivatives corresponding to one or more aminoacidic modifications. The A:12-21peptide SLYQLENYCN (Nt-INSP-3) was oxidized to the oxPTM-INSP-3 derivatives SL-DOPA-QLENY-Cysteate-N and SL-DOPA-QLEN -DOPA-Cysteate-N; the B:21-30 peptide ERGFFYTPKT (Nt-INS-P6) was oxidized to the oxPTM-INSP-6 derivatives ERGYYYTPKT, ERGYY-DOPA-TPKT, and ERGYYYTPKTR. Autoantibody reactivity to the Nt-INSP and oxPTM-INS peptides was tested using sera from 63 subjects with new-onset type 1 diabetes (T1D) (Linkoping and IMDIAB biobanks). In a second cohort we evaluated, in parallel, $CD4^+$ and $CD8^+$ T-cell and autoantibody responses to the oxPTM-INS peptides derivatives by using fresh PBMCs and sera collected from 18 recent onset and 5 additional established T1D patients.

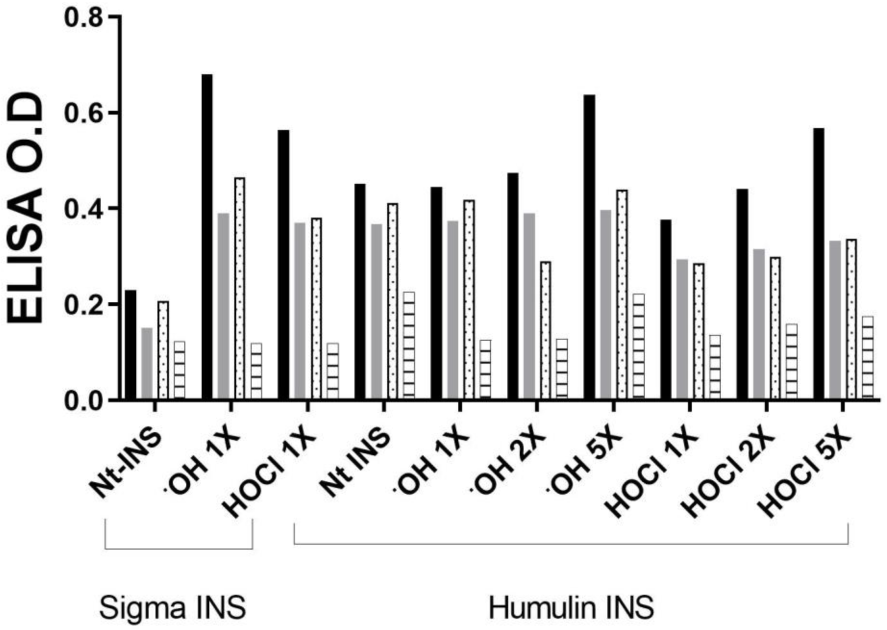

ESM Figure 2. Reactivity pattern of type 1 diabetes samples to oxPTM Humulin R® insulin versus Sigma oxPTM-INS. We show reactivity for three type 1 diabetes (T1D) patients (black, gray and dotted bars) and compared it to one healthy control (strike pattern). The term 1x•OH represent exposure of insulin to 4.5 mM $CuCl_2$ and 9 mM hydrogen peroxide (modification introduced by the Fenton reaction). 1x HOCl means exposure to 2mM HOCl. Different oxidation conditions were tested to optimized for Humulin R® insulin that was formulated differently by the manufacturer. The terms "2x " and "5x " denotes the scaled-up of the above treatment conditions. Humulin R® oxPTM displays similar reactivity as for the Sigma insulin.

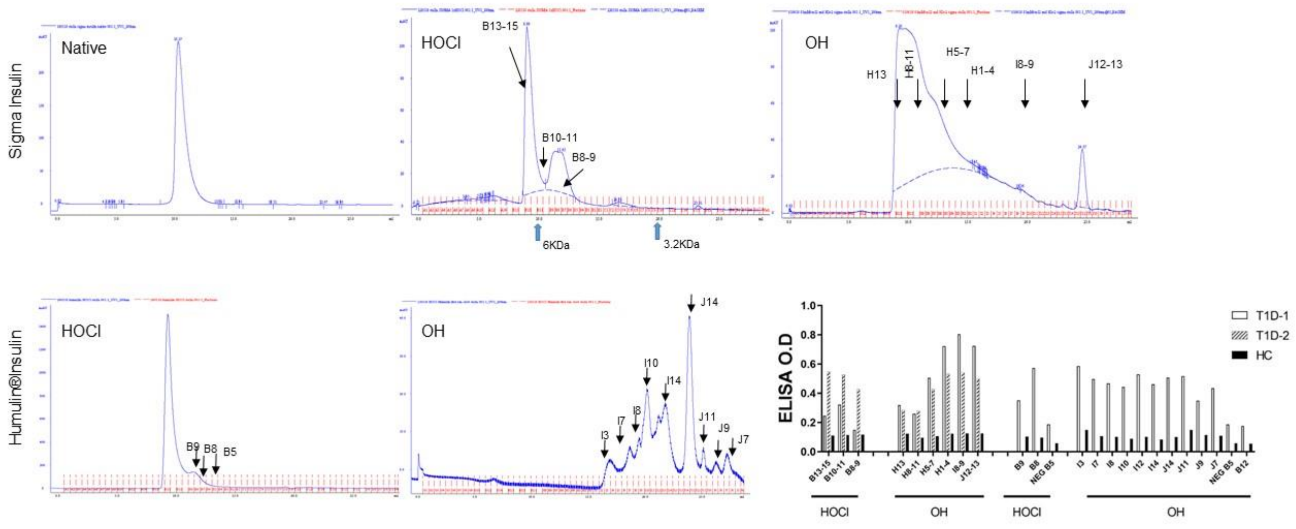

ESM Figure 3. The size exclusion chromatography ÄKTA. Superdex 30 column suitable for the detection of low molecular weights (100-7000 kDa) was used for separating the various insulin fragments that were obtained after the oxidative posttranslational modification (oxPTM) of either Sigma insulin or Humulin R® insulin. Sigma and Humulin R® native insulin (Nt-INS) display a single peak at around 10 ml. Following oxidation, oxPTM-INS different small (< 6kDa) insulin fragments were observed. Sigma insulin exposed to HOCl showed a major peak at around 10 ml with additional peak at lower molecular weight. Exposing Sigma insulin to •OH resulted in a broad peak of small molecular fragments < 6 kDa at elution volumes between 10 ml and 25 ml, and an additional peak at 25 ml. Humulin R® insulin, subjected to oxidation with HOCl displayed a major peak at 10 ml with an additional shoulder of a smaller molecular weight. For •OH modification of Humulin R® insulin, the major native peak corresponding to native insulin disappeared but instead we observed a set of multiple peaks towards the end of the chromatograph corresponding to small molecular weights < 6 kDa. **c.** Type 1 diabetes (T1D) serum samples but not healthy controls (HC) bound to the small molecular weight fractions that resulted from either HOCl or •OH modification.

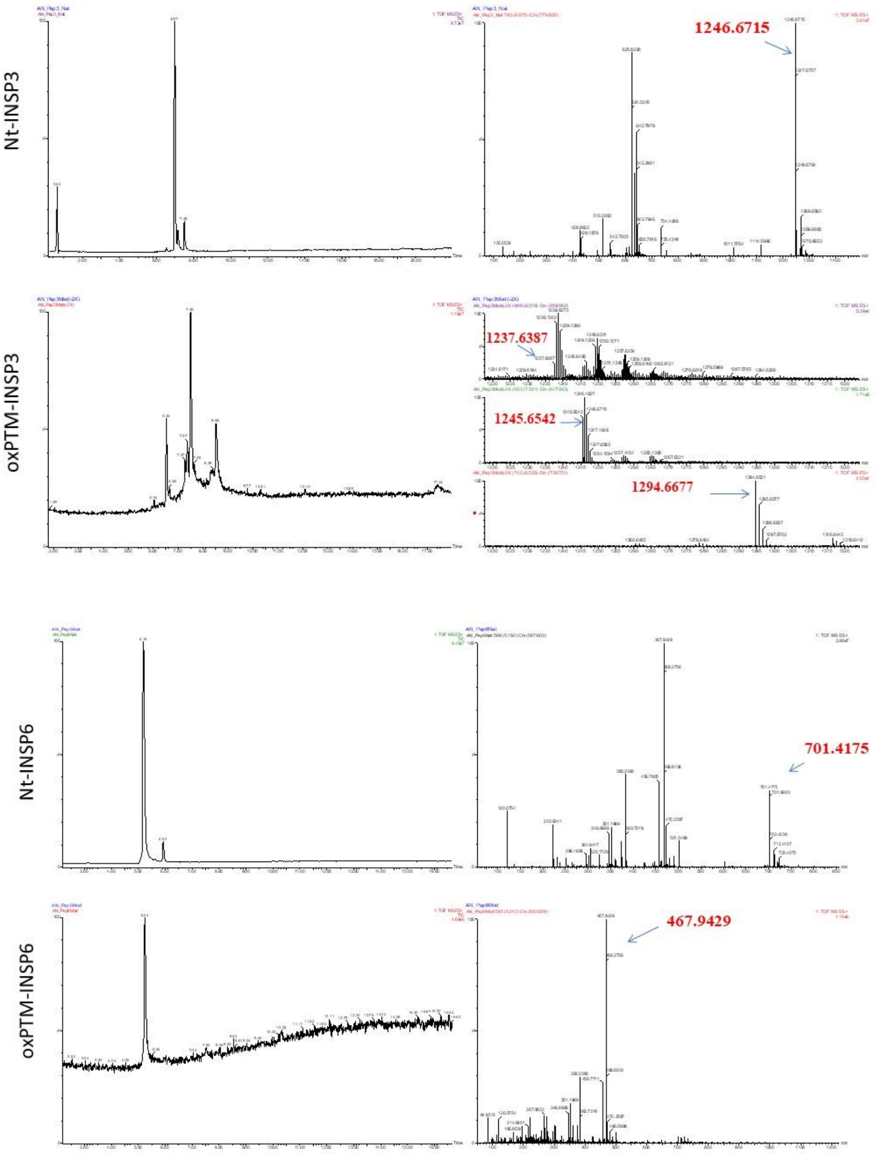

ESM Figure 4. Testing peptides oxidation by TOF-MS/ES+TIC. Peptide candidates were exposed to either HOCl or •OH and were assessed by TOF-MS/ES+TIC. Native insulin peptide 3 (Nt-INSP3) display a single ionization peak corresponding to SLYQLENYCN [R-NH3]+, m/z 1246.6751. oxPTM-INSP-3 corresponding to m/z 1237.6387, 1245.6542 and 1294.6677 a shift in mass as an indication of oxidative modification (potentially corresponding to addition of single or multiple OH (16Da). Native insulin peptide 6 (Nt-INSP6, ERGFFYTPKTR, m/z 1401.57) display a single LC-MS/MS peak at 701.4175 corresponding to double ionization mass. After oxidation, there was significant degradation and only a single peak of 467.9429 m/z is seen.

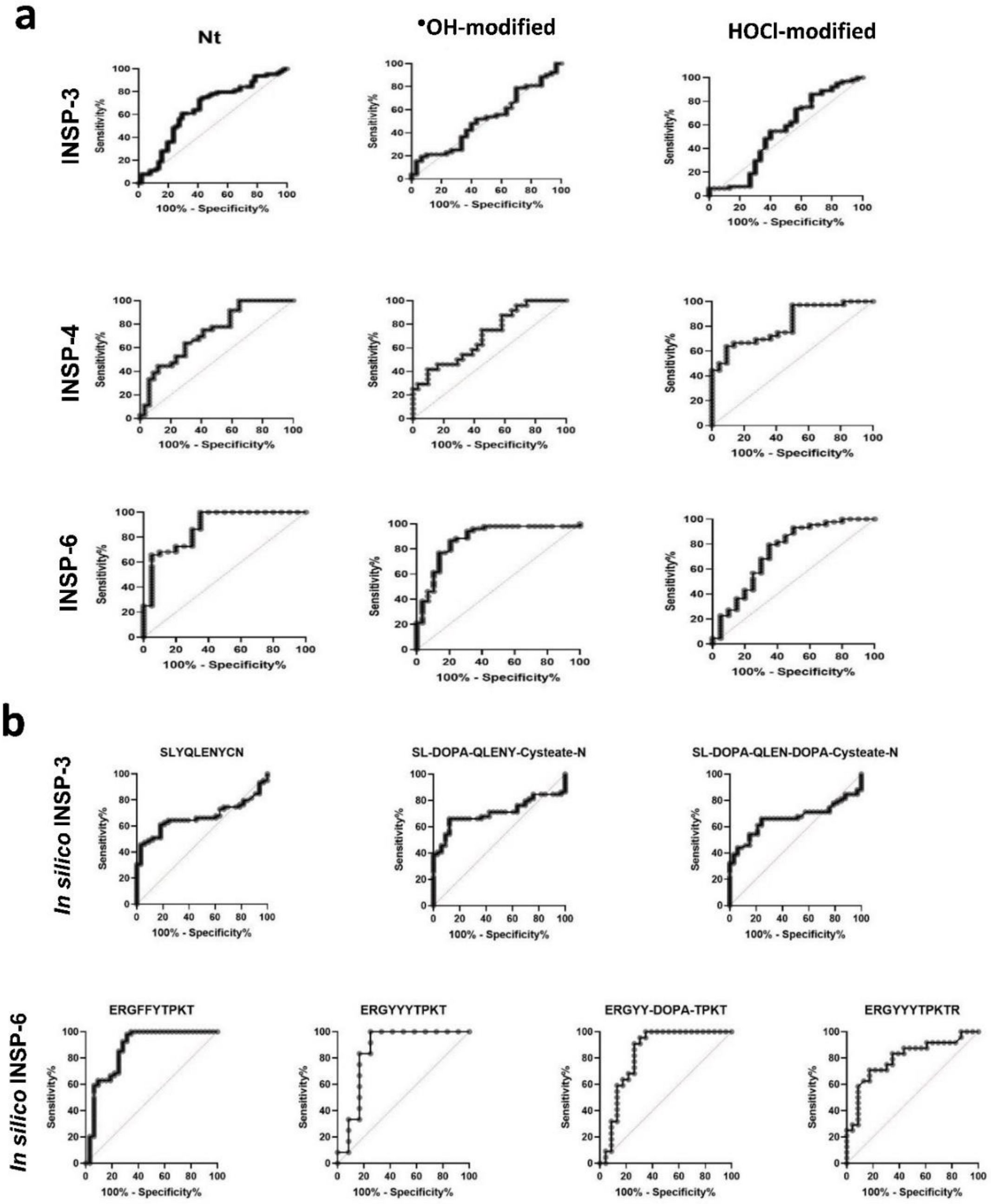

ESM Figure 5. ROC Curve analysis for insulin peptides INSP-3, INSP-4 and INSP-6, native and modified *in house* (a) or *in silico* (b). The highest specificity/sensitivity observed is for peptide 6 whether it was oxidized *in house* by •OH or HOCl, or *in silico* oxidized, which showed ROC-AUC>0.8 for all peptide versions.

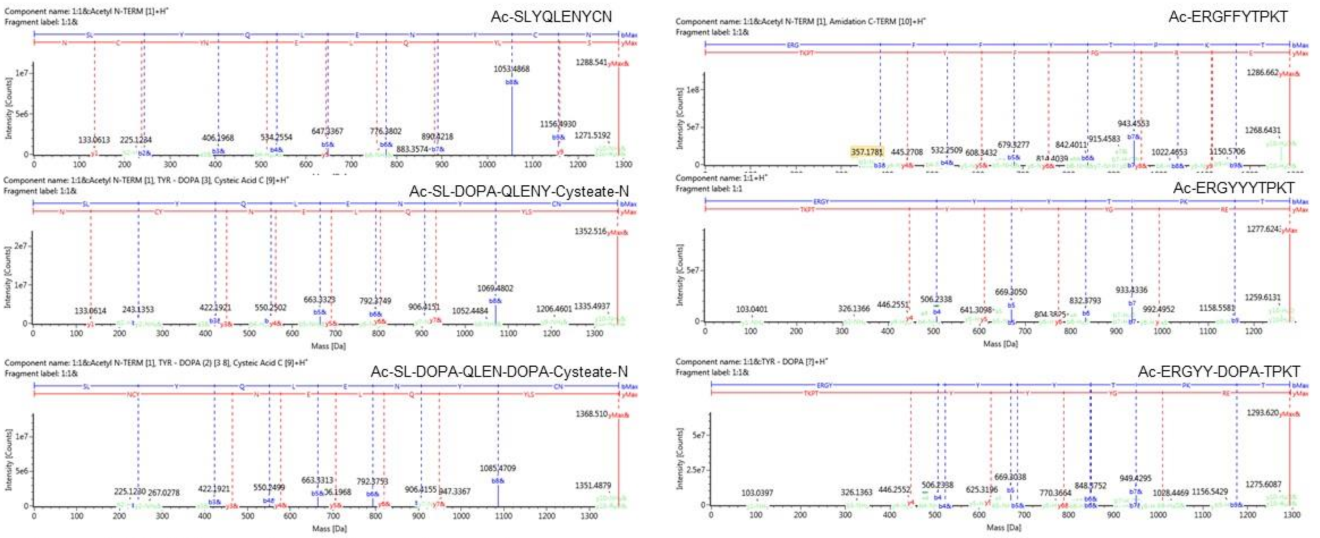

L	225.123	L		L	
Y	406.197	DOPA	422.192	DOPA	422.192
Q	534.256	Q		Q	
L	647.34	L		L	
E	776.382	E		E	
N	890.425	N		N	
Y	1053.49	Y		DOPA	1085.48
C	1156.5	Cys		Cy	
N	1288.55	N	1352.53	N	1368.53

R		R		R	
G	357.188	G		G	
F	532.251	Y	669.299	Y	
F	679.32	Y	832.362	Y	
Y	842.383	Y		DOPA	949.405
T	943.431	T		T	
P	1022.47	P		P	
K	1150.57	K		K	
T	1286.65	T	1277.62	T	1293.61

ESM Figure 6. Peptide sequence confirmation by UPLC-qTOF/MS^{e}. Fragmentation spectra of the three versions of peptides 3 (INSP-3) and 6 (INSP-6) is shown. INSP-3 has common sequence backbone, SLYQLENYCN [R-NH3]$^{+}$, m/z 1288.551), Ac-SL-DOPA-QLENY-cysteate-N; [R-NH3]$^{+}$, m/z 1352.5162) and Ac-SL-DOPA-QLEN-DOPA-cysteate-N; [R-NH3]$^{+}$, m/z 1368.5091). The fragmentation pattern of native INSP-6 and oxPTM-INSP6, ERGFFYTPKT- NH2; [R-NH_3]$^{+}$, m/z 1286.6626); ERGYYYTPKT; [R-NH3]+, m/z 1277.6253) and ERGYY-DOPA-TPKT; [R-NH3]+, m/z 1293.6213 is also shown. Table highlights the fragment ions identified with different m/z as a result of modification.

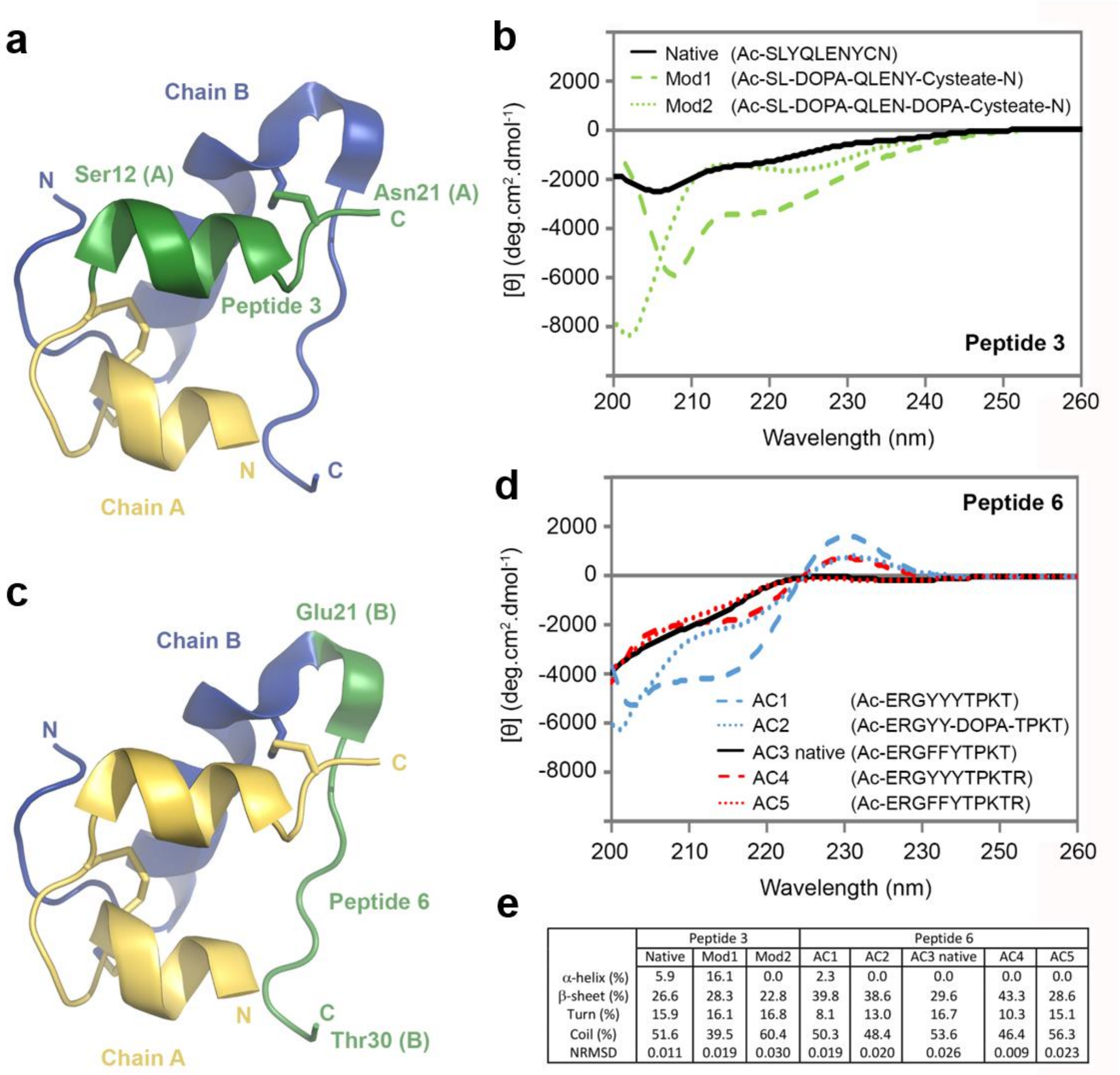

	Peptide 3			Peptide 6				
	Native	Mod1	Mod2	AC1	AC2	AC3 native	AC4	AC5
α-helix (%)	5.9	16.1	0.0	2.3	0.0	0.0	0.0	0.0
β-sheet (%)	26.6	28.3	22.8	39.8	38.6	29.6	43.3	28.6
Turn (%)	15.9	16.1	16.8	8.1	13.0	16.7	10.3	15.1
Coil (%)	51.6	39.5	60.4	50.3	48.4	53.6	46.4	56.3
NRMSD	0.011	0.019	0.030	0.019	0.020	0.026	0.009	0.023

ESM Figure 7. Structural changes in the neo-antigenic insulin peptides. a. Structure of human insulin (PDB ID: 5bts) highlighting the location of peptide 3 in chain A (coloured green). b. Circular dichroism spectra for native, SL-DOPA-QLENY-cysteate-N (Mod1) and SL-DOPA-QLEN-DOPA-cysteate-N (Mod 2) forms of peptide 3. **c.** Structure of human insulin highlighting the location of peptide 6, Ac-ERGFFYTPKT within chain B (coloured green). d. Circular dichroism spectra of native Ac-ERGFFYTPKT(AC3) and Ac-ERGFFYTPKTR (AC5) versus oxPTM-INSP-6 ERGYYYTPKT (AC1), ERGYY-DOPA-TPKT (AC2) or ERGYYYTPKTR (AC4). **e.** Secondary structure analysis of the circular dichroism data using BeStSel implies significant structural changes of peptides upon oxPTM but which are not correlated with the degree of modification.

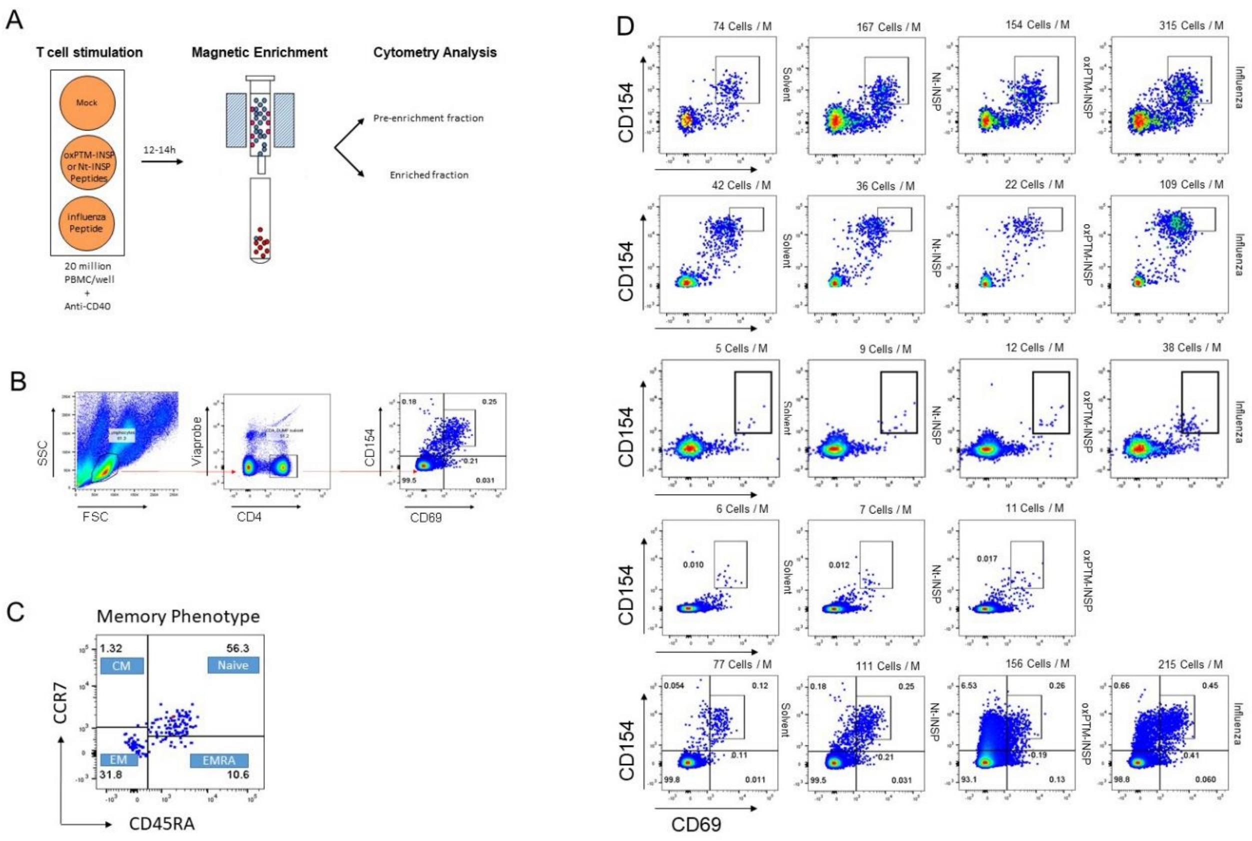

ESM Figure 8. Strategy for CD154 based detection of CD4+ T cell responses. (A) The surface marker upregulation assay consists of a short-term activation of PBMC with solvent only (Mock) or specific peptides of interest (oxPTM-INSP, Nt-INSP, or an influenza peptide) in individual wells for 10-14 hours in the presence of an anti-CD40 blocking antibody to prevent downregulation of CD154. The recently activated CD154+ cells are then magnetically labeled and then enriched over an MS column, reserving a pre-enriched fraction to estimate the total number of CD4+ T cells in the sample. Both the pre-enriched and enriched samples are then analyzed by flow cytometry. (B) The gating scheme for the CD154 assay is to select lymphocytes on a forward scatter (FSC) versus side scatter (SSC) plot, select live CD4+ T cells on a CD4 versus Viaprobe plot, and then select and count CD154+CD69+ cells as the recently activated antigen specific population. (C) A biaxial plot of CD45RA versus CCR7 was gated based on total CD4+ T cells and the utilized to partition CD154+ cells into naïve (CD45RA+CCR7+), TCM (CD45RA-CCR7+), TEM (CD45RA-CCR7-), and TEMRA (CD45RA+CCR7-) phenotypes. (D) Surface marker upregulation results obtained for five subjects with T1D are all shown. In some subjects, the frequency of CD154+CD69+ (epitope specific) T cells was higher for native insulin peptides, oxidized insulin peptides, and the influenza peptide than the solvent only control

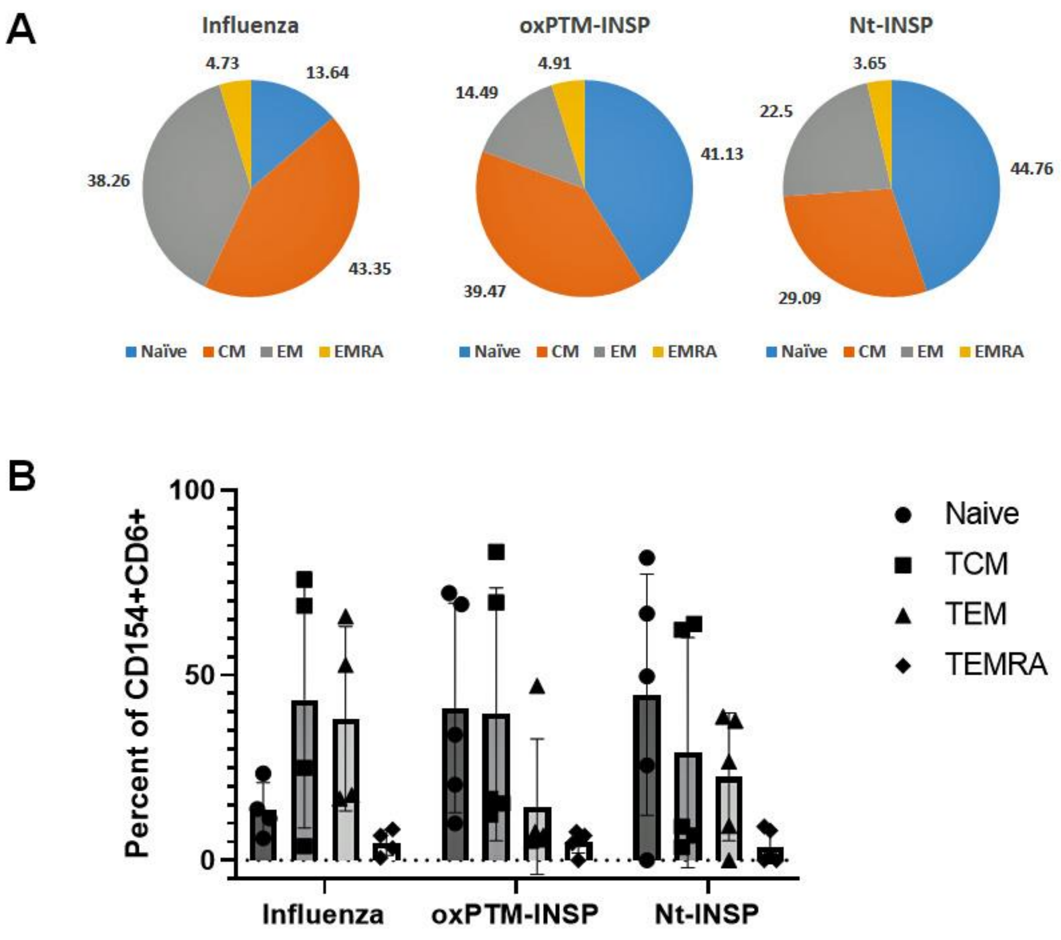

ESM Figure 9. CD4+ T cell responses toward native and oxidized insulin peptides. Across the five T1D subjects assayed, we utilized surface staining for CD45RA and CCR7 on CD154+CD69+ T cells to classify epitope specific T cells as Naïve (CD45RA+CCR7+), TCM (CD45RA-CCR7+), TEM (CD45RA-CCR7-) or TEMRA (CD45RA+CCR7-). (A) Averaging across the five subjects, influenza specific T cells were predominantly TCM and TEM as expected. Native insulin and oxidized insulin specific T cells trended toward having a higher percentage of naïve cells (44.7% and 41.1% respectively) but also had notable proportions of TCM and TEM, suggesting that there is an existing pool of memory T cells that recognizes these insulin peptides in subjects with T1D. (B) Plotting the same data as individual data points for each subject preserved the same tendencies, but reveals some heterogeneity in the relative proportion of Naïve, TCM, and TEM cells.

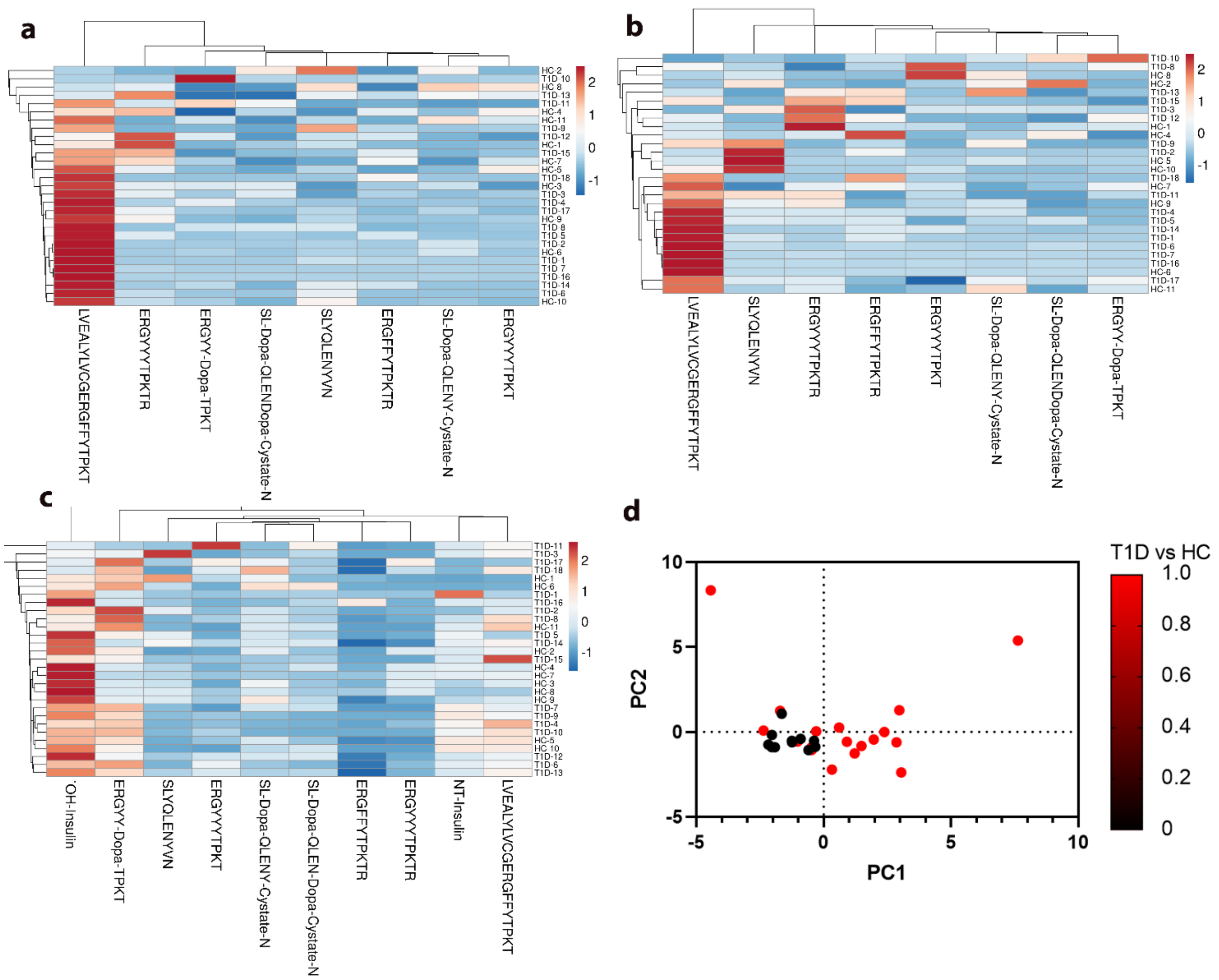

Figure 10. Hierarchical cluster analysis. Hierarchical cluster analysis and principle component analysis (PCA) revealed association between the responses to different oxPTM-INSP and identify clustering of type 1 diabetes versus healthy control samples. We observed association between Nt-INSP-4 and ox-PTM-INS-P6, ERGYYYTPKTR for CD4^{+} (a) and CD8^{+} (b). For CD4^{+} response Nt-INSP-4 is also closely associated to ERGYY-DOPA-TPKT. For IgG response (c) ERGYY-DOPA-TPKT is associated with $^{\bullet}$OH -Insulin. We also observed clustering of response of type 1 diabetes samples using PCA analysis of all responses, CD4, CD8 and IgG (d). We observed cluster of 11 type one diabetes samples with PC1>0 while the rest clustered with healthy control samples with PC1<0.
